# RNAi Dynamics in Juvenile *Fasciola* spp. Liver Flukes Reveals the Persistence of Gene Silencing *In Vitro*

**DOI:** 10.1371/journal.pntd.0003185

**Published:** 2014-09-25

**Authors:** Paul McVeigh, Erin M. McCammick, Paul McCusker, Russell M. Morphew, Angela Mousley, Abbas Abidi, Khalid M. Saifullah, Raman Muthusamy, Ravikumar Gopalakrishnan, Terry W. Spithill, John P. Dalton, Peter M. Brophy, Nikki J. Marks, Aaron G. Maule

**Affiliations:** 1 Molecular Biosciences: Parasitology, The Institute for Global Food Security, School of Biological Sciences, Queen's University Belfast, Medical Biology Centre, Belfast, United Kingdom; 2 Institute of Biological, Environmental and Rural Sciences, Aberystwyth University, Aberystwyth, Wales, United Kingdom; 3 Department of Zoology, Aligarh Muslim University, Aligarh, Uttar Pradesh, India; 4 Tamil Nadu Veterinary and Animal Sciences University, Chennai, Tamil Nadu, India; 5 AgriBio, the Centre for AgriBioscience, School of Life Sciences, LaTrobe University, Melbourne, Australia; Tufts University, Cummings School of Veterinary Medicine, United States of America

## Abstract

**Background:**

*Fasciola* spp. liver fluke cause pernicious disease in humans and animals. Whilst current control is unsustainable due to anthelmintic resistance, gene silencing (RNA interference, RNAi) has the potential to contribute to functional validation of new therapeutic targets. The susceptibility of juvenile *Fasciola hepatica* to double stranded (ds)RNA-induced RNAi has been reported. To exploit this we probe RNAi dynamics, penetrance and persistence with the aim of building a robust platform for reverse genetics in liver fluke. We describe development of standardised RNAi protocols for a commercially-available liver fluke strain (the US Pacific North West Wild Strain), validated via robust transcriptional silencing of seven virulence genes, with in-depth experimental optimisation of three: cathepsin L (FheCatL) and B (FheCatB) cysteine proteases, and a σ-class glutathione transferase (FheσGST).

**Methodology/Principal Findings:**

Robust transcriptional silencing of targets in both *F. hepatica* and *Fasciola gigantica* juveniles is achievable following exposure to long (200–320 nt) dsRNAs or 27 nt short interfering (si)RNAs. Although juveniles are highly RNAi-susceptible, they display slower transcript and protein knockdown dynamics than those reported previously. Knockdown was detectable following as little as 4h exposure to trigger (target-dependent) and in all cases silencing persisted for ≥25 days following long dsRNA exposure. Combinatorial silencing of three targets by mixing multiple long dsRNAs was similarly efficient. Despite profound transcriptional suppression, we found a significant time-lag before the occurrence of protein suppression; FheσGST and FheCatL protein suppression were only detectable after 9 and 21 days, respectively.

**Conclusions/Significance:**

In spite of marked variation in knockdown dynamics, we find that a transient exposure to long dsRNA or siRNA triggers robust RNAi penetrance and persistence in liver fluke NEJs supporting the development of multiple-throughput phenotypic screens for control target validation. RNAi persistence in fluke encourages *in vivo* studies on gene function using worms exposed to RNAi-triggers prior to infection.

## Author Summary

RNA interference (RNAi) is a method for selectively silencing (or reducing expression of) mRNA transcripts, an approach which can be used to interrogate the function of genes and proteins, and enables the validation of potential targets for anthelmintic drugs or vaccines, by investigating the impact of silencing a particular gene on parasite survival or behaviour. This study focuses on liver fluke parasites, which cause serious disease in both humans and animals. We have only a handful of drugs with which to treat these infections, to which flukes are developing resistance, and no anti-fluke vaccines have yet been developed. New options for treatment and control of liver fluke parasites are sorely needed, and RNAi is a powerful tool in the development of such treatments. This study developed a set of simple methods for triggering RNAi in juvenile liver fluke, which show that although robust transcriptional suppression can be readily achieved across all targets tested, protein suppression occurs only after a target-specific lag period (likely related to protein half-life), which may require >25 days under current *in vitro* maintenance conditions. These findings are important for researchers aiming to employ RNAi in investigations of liver fluke biology and target validation.

## Introduction

*Fasciola* spp. liver flukes are the causative agents of fascioliasis, or liver fluke disease. Their growing importance as human neglected tropical disease pathogens, alongside their impact on animal health, welfare and global food security, is recognised [1]–[3]. This pathology is compounded by established incidences of field resistance to the handful of available flukicidal drugs in both veterinary and human infections [4], [5]. Although adult fluke are the reproductively active stage and cause physical-, nutritional- and immuno-pathology to the vertebrate host, it is the tissue-penetrating invasive newly-excysted juvenile (NEJ)/juvenile stage *Fasciola*, that cause significant damage to host tissues during their migration from the gut lumen, through the hepatic parenchyma to the bile ducts. These NEJs are not adequately targeted by existing chemotherapy; triclabendazole is the only flukicide with significant activity against juvenile liver fluke [6]. Novel flukicidal treatment options are urgently required, especially those with activity against juvenile fluke.

Beyond two initial reports of RNA interference (RNAi) [7], [8], there have been no documented accounts of efforts to develop functional genomics tools for the study of liver fluke biology and the validation of new drug and vaccine targets. Traditionally, such studies were hindered by a relative lack of liver fluke nucleotide sequence datasets compared to other parasitic helminths. However, ongoing efforts have seen the primarily adult-derived sequence datasets available through both GenBank and the Wellcome Trust Sanger Institute complemented by the public release of transcriptome datasets for adult specimens of both temperate and tropical liver fluke (*F. hepatica* and *F. gigantica* respectively), soon to be complemented by juvenile transcriptome datasets and draft *F. hepatica* genome sequences [9]–[12]. A suite of functional genomics tools and associated functional assays will be required in order to effectively exploit these sequence resources.

RNAi permits the destruction of target mRNA, and subsequent suppression of target protein, by the introduction of double stranded (ds)RNA trigger molecules of complementary sequence to an mRNA target [13]. In parasitic helminths and other species that are difficult or impossible to transform by available molecular genetic methods, RNAi provides a means of investigating gene function through the relatively simple generation of organisms in which the expression of a target gene has been reduced or ablated. This approach permits both the testing of basic biological hypotheses, and the identification of deleterious phenotypes that might inform drug/vaccine target validation efforts. While parasitic flatworms appear to be broadly amenable to RNAi [14]–[16], the initial development of RNAi methodology in any species requires optimisation of experimental variables, sometimes on a target-by-target basis, validated by measurement of the extent and specificity of transcript and protein knockdown [17]. *Schistosoma mansoni* has been well served by studies describing extensive experimental optimisations of RNAi methodology [18]–[22], enabling the effective exploitation of functional genomics tools by the associated research community. RNAi can be induced in *F. hepatica* by either soaking or electroporation [7], [8]. Considering the former method to be less onerous and less technically demanding, and therefore more likely to be employed by the research community in both small- and large-scale assay systems, here we set out to apply dsRNA exposure-induced RNAi to silence a range of virulence gene targets (and proposed vaccine antigen candidates), including cathepsin B and L cysteine proteases, fatty-acid binding protein, leucine aminopeptidase, and μ-, ω-, and σ-class glutathione transferases, in NEJs of a commonly-used, commercially-available *F. hepatica* isolate (US Pacific North West Wild Strain). Finding that RNAi transcript, protein and phenotype dynamics were quite distinct in this system compared to those reported previously in Oberon and Fairhurst isolates [8], we performed a set of in-depth experimental optimisations in order to characterise the RNAi mechanisms operating in this liver fluke isolate. Focusing on three examples of commonly cited virulence genes and vaccine targets – cathepsin B (FheCatB), cathepsin L (FheCatL) and a sigma-class glutathione transferase (FheσGST), we have systematically investigated the effects of variations in dsRNA trigger concentration, experimental timecourse, and dsRNA soak exposure protocol using both long (200–220 nt) dsRNA and 27 nt short interfering (si)RNA triggers, on knockdown of both target transcript and protein. We show that, although apparently operating in a manner distinct to that reported in existing literature [8], robust, persistent RNAi is eminently achievable in NEJs of the *F. hepatica* Pacific North West Wild strain, and in the tropical liver fluke *F. gigantica*, using a simple dsRNA-exposure protocol.

## Methods

### Parasites

*F. hepatica* (US Pacific North West wild strain) metacercariae, with outer cyst wall removed, were obtained from Baldwin Aquatics Inc (Monmouth, Oregon, USA), and stored at 4°C until required. *F. gigantica* metacercariae were obtained from naturally infected wild snails collected in Aligarh, Uttar Pradesh, India, by researchers at Aligarh Muslim University. Outer cyst walls were removed by incubation in a solution of 1% pepsin, 4 mM HCl, for 90 min at 37°C, followed by several washes in distilled water. Metacercariae were then excysted by incubation in 0.6% sodium bicarbonate, 0.45% sodium chloride, 0.4% sodium tauroglycocholate, 0.025 M HCl, 0.4% L-Cysteine, for up to 3 h at 37°C. After approximately 75 min, at 10–15 min intervals, NEJs were transferred to RPMI 1640 without phenol red (Life Technologies), in which they were maintained at 37°C for a maximum of 3 h until transferred to soaking media. *F. gigantica* cysts were exposed to excystment media for 3 h, after which they were transferred to RPMI for incubation, in which their excystment completed within 18 h. Variations in maintenance/soaking conditions are described below.

### RNAi methodology

Experimental and negative control (dsCTRL) RNAi triggers comprised “long” dsRNA molecules (sized between 200–320 nt) generated by T7 polymerase-driven transcription of single RNA strands (MEGAshortscript T7 Kit, Ambion) from target-specific PCR product templates labelled either sense or antisense with a T7 RNA polymerase promoter sequence (5′- TAATACGACTCACTATAGGGT -3′). Primers used to generate long dsRNA template PCR products were: FheCatB2 [U58000]: forward, 5′- GTTGTCAGCCCTGGATGTTT -3′, reverse, 5′ -GTCCTGGAATATGGCGAAAG- 3′; FheCatL [designed for maximum similarity against alignment of all FheCatL clades on GenBank, see Figure S1]: forward, 5′-TKRTTATGTGACGGAGGTGA-3′, reverse, 5′-GCCBKRTAHGGRTAAK-3′; dsCTRL [bacterial neomycin phosphotransferase, U55762]: forward, 5′- GGTGGAGAGGCTATTCGGCTA -3′, reverse, 5′- CCTTCCCGCTTCAGTGACAA -3′; FheFABP [AJ250098]: forward, 5′- TAAGATTACAACTTTCACATTTGGC -3′, reverse, 5′- GCGGGATGCTCAAAATCCGTC -3′; FheLAP [AY644459]: forward, 5′- GGTAGTGAACTGTCAATTGTTCCG -3′, reverse, 5′- GAGTAGCAGATGTTTGCGTTGC -3′; FheμGST [designed against alignment of M77682, M93434, M77680, M77681, M77679, see Figure S2]: forward, 5′- ACACCCGAGGAACGAGCTCG -3′, reverse, 5′- TCGTAAACCATAAAGTCCACATGG -3′; FheωGST [JX157880]: forward, 5′- GATTGGTATCTGGAGTTATATCCG -3′, reverse, 5′- AATAAAATACCATGCGATTGAGCC -3′; FheσGST [DQ974116]: forward, 5′- AATTCGCCTTCTGCTCACTTGC -3′, reverse, 5′- GTAATACTCTTCGTCTGTTTCACC -3′). Endogenous templates were amplified from *F. hepatica* (or *F. gigantica*) cDNA as appropriate; due to high identity between the relevant orthologues, we were able to use *F. hepatica* primers to amplify FgigσGST and FgigGAPDH from *F. gigantica* (see ‘quantification of transcript knockdown’ for GAPDH primer sequences). These amplicons were sequence confirmed. Exogenous dsCTRL templates were amplified from an eGFP plasmid (Promega). Complementary single-stranded RNAs were combined and annealed (75°C for 5 min, 19°C for 30 min) before treatment with DNase (Turbo DNase, Life Technologies) and RNase (RNase If, New England Biolabs) and subsequent phenol/chloroform extraction and ethanol precipitation. Pelleted dsRNA was briefly air-dried (∼10 min) at room temperature, resuspended in ≥100 µl DEPC-treated H_2_O, and stored at −20°C. All dsRNAs were quantified using a NanoDrop ND1000 Spectrophotometer and analysed for the presence of a discrete, correctly sized product on a non-denaturing 1% (w/v) agarose TAE gel. Only dsRNAs that gave a discrete correctly sized band, and 260/280 and 260/230 ratios >1.8 were used in RNAi experiments. Typical dsRNA concentrations following synthesis were in excess of 2000 ng/µl. Verified sizes of dsRNAs were: dsFheCatB: 248 nt; dsFheCatL 246 nt: nt; dsCTRL: 223 nt; dsFABP: 313 nt; dsFheLAP: 234 nt; dsFheμGST: 218 nt; dsFheωGST: 209 nt; dsFheσGST: 223 nt.

“Dicer substrate” 27mer siRNAs to FheσGST were designed and synthesised by Integrated DNA technologies (IDT; www.idtdna.com) (target sites: siGST1: 5′- TTCGAGGACTATCAATTCACAATGGAT -3′; siGST2: 5′- GGGAAACTTAGACGTTATCAAGAATCG -3′; siGST3: 5′- ATTGCTCGATTGCTTGCCAGACAATTC -3′; IDT's off the shelf DS Scrambled Neg siRNA (5′- CTTCCTCTCTTTCTCTCCCTTGTGA -3′) was used as the siCTRL). Upon receipt, siRNAs were resuspended in the supplied buffer to 100 µM concentration, and stored at −80°C in single-use aliquots until required.

NEJs were soaked in solutions of long dsRNA or siRNA dissolved to defined concentrations in RPMI 1640, in 2 ml round-bottomed microcentrifuge tubes. Soaks were handled at least in triplicate alongside triplicate untreated (no dsRNA) and size- and concentration-matched negative control long dsRNA (dsCTRL)- or negative control siRNA (siCTRL)-treated controls. For qPCR experiments 20 NEJs were used per soak, western blot experiments used 50 NEJs per soak. Soaks were performed under one of five variations on a dsRNA exposure protocol (described in Results – Burst exposure to trigger). Unless specified otherwise, NEJs were maintained aseptically in 1 ml RPMI 1640, at 37°C in a 5% CO_2_ atmosphere. RPMI was replaced at 2–3 day intervals for periods of up to 25 days. Worms were visually assessed for aberrant motility or morphology during media changes. At the end of each soak experiment, worms were either processed for RNA/protein extraction immediately, or snap frozen and stored at −80°C for processing at a later time.

### Quantification of transcript knockdown

mRNA was extracted from NEJs using the Dynabeads mRNA Direct kit (Life Technologies). We employed the mini extraction protocol as directed by the manufacturer (except that all washes were performed with only 300 µl buffer), and eluted in 12.5 µl Tris-HCl. Eluted mRNA was DNase digested using the Turbo DNA Free kit (Life Technologies) in a total volume of 15 µl, from which 8 µl was added directly to a 20 µl reverse transcription reaction using Applied Biosystems' High Capacity RNA to cDNA Kit, as described by the manufacturer. Following reverse transcription, cDNA was diluted with an equal volume of water before use in qPCR. Triplicate qPCR reactions were performed in 10 µl reaction volumes using the FastStart SYBR Green Master mix (Roche Applied Science), containing primers (FheCatB: forward, 5′-GCACGTACTGTGGTCAGGGGTG-3′, reverse, 5′-GTCCTGGAATATGGCGAAAG-3′; FheCatL: forward, 5′-TKRTTATGTGACGGAGGTGA-3′, reverse, 5′-GTATAGAAGCCAGTCACTTTGGC-3′; FheFABP: forward, 5′- AACAAAATGACTATTAAAATGG -3′, reverse, 5′- GCGGGATGCTCAAAATCCGTC -3′, GAPDH reference amplicon [AY005475]: forward, 5′- GGCTGTGGGCAAAGTCATTC -3′, reverse, 5′- AGATCCACGACGGAAACATCA -3′; FheLAP: forward, 5′- GGTAGTGAACTGTCAATTGTTCCG -3′, reverse, 5′- GGCAGATAGAGTGCATGGTACG -3′; FheμGST: forward, 5′- ACACCCGAGGAACGAGCTCG -3′, reverse, 5′- GGATAAGCATGGAATGCTTGGT -3′; FheωGST: forward, 5′- GATTGGTATCTGGAGTTATATCCG -3′, reverse, 5′- ATACGCAGACGCATTAGCTTCC -3′; FheσGST: forward, 5′- AATTCGCCTTCTGCTCACTTGC -3′, reverse, 5′- TCTTCACACTCACCAATGATACG -3′) at final concentrations of either 200 nM (FheCatB, FheCatL) or 1500 nM (FheσGST), as well as 2 µl cDNA template (or H_2_O in the case of negative PCR controls). As above, *F. hepatica* primers were used to amplify FgigσGST and FgigGAPDH from *F. gigantica*. Amplification was performed on a Qiagen RotorGene Q 5-plex HRM instrument (10 min 95°C; 40 cycles: 10 sec, 95°C, 15 sec, 60°C, 30 sec, 72°C, followed by melt curve analysis of amplicons). Relative expression analysis was performed manually using Pfaffl's Augmented ΔΔCt method [23] (which normalises expression in each sample relative to the untreated control, standardised to a GAPDH reference amplicon), with amplification efficiencies of individual reactions calculated using Real Time PCR Miner software (http://ewindup.info/miner/data_submit.htm; [24]). This analysis method produces a ratio of target transcript change relative to the untreated control (i.e. where a value of 1.0 represents no change) ± SEM. These ratios are plotted in bar graphs in the qPCR figures. Statistical analyses were performed on the ratios produced following relative expression analysis, using one-way ANOVA with Tukey's post test. Statistical significance was determined relative to the effects of negative control treatments (dsCTRL or siCTRL) on target gene expression.

### Quantification of protein knockdown

Following soaking/maintenance using Protocol IV (as described in Results – Burst exposure to trigger), 50 NEJs were ground under liquid nitrogen in a round-bottom 2 ml microcentrifuge tube, after which 50 µl radioimmunoprecipitation assay (RIPA) buffer, containing protease inhibitors (Complete Mini, Roche Applied Science) and phosphatase inhibitors (Phosphatase Inhibitor Cocktail 2, Sigma Aldrich), was added. Tubes were then subjected to 3× 20 sec cycles of sonication, and left on ice for 60 min before quantification of protein content by BCA assay (BCA Protein Assay Kit, Pierce). Following addition of 50 µl 2× Novex Tricine SDS Sample Buffer (Life Technologies), and *tris*(2-carboxyethyl)phosphine (TCEP) to a final concentration of 10 mM, samples were denatured at 95°C for 5 min, and ≤25 µl of each sample (equalised for protein content) loaded into wells of a pre-cast 10–20%, 10 well, 1.0 mm, Tricine Gel (Life Technologies), alongside one lane containing 15 µl SeeBlue Plus2 Prestained Standard (Life Technologies). Gels were run in an Xcell Surelock Mini-Cell apparatus (Life Technologies) at 135 V for 45–60 min in tricine running buffer, before electroblotting onto nitrocellulose membrane in an Xcell Mini-Cell apparatus (Life Technologies), at 35 V, for 2 h. Membranes were washed briefly with TBST (Tris-Buffered Saline prepared from tablets [Sigma Aldrich], containing 0.05% Tween 20) before incubation in blocking buffer (TBST containing 5% Blotting Grade Non-Fat Dry Milk [Biorad]), for 30 min, at room temperature, with rotation. Membranes were then probed overnight at 4°C, with rotation, in blocking buffer containing two primary antisera (one target and one loading control normaliser) (rabbit-anti-σGST 1/15,000; rat-anti-FheCatB2 1/1000; sheep-anti-FheCatL1, 1/2000; rabbit-anti-actin [20]–[33, Sigma Aldrich] 1/400). Following 5×5 min washes in TBST, membranes were probed with appropriate secondary antisera (alkaline-phosphatase conjugated goat-anti-rabbit (Invitrogen), goat-anti-rat (Invitrogen), or donkey-anti-sheep [Sigma Aldrich], diluted 1/1000 in blocking buffer), for 2 h at room temperature. Following 5×5 min washes in TBST, membranes were exposed with NBT/BCIP solution (prepared from tablets, Roche Applied Science). Development, carefully monitored by eye, occurred in as little as 20 sec and was terminated by washing the membrane thoroughly in distilled H_2_O. Membranes were then dried, scanned, and band intensities quantified by densitometry using ImageJ software (http://rsbweb.nih.gov/ij/). In order to permit relative protein quantitation, band intensities for the proteins of interest were normalised relative to the intensity of the loading control band from the same sample, and then this figure was expressed as a percentage relative to the untreated control ( = 100%) sample. Statistical analyses were performed using one way ANOVA with Tukey's post test. All statistical significances presented in figures are expressed relative to the dsCTRL treatment.

### Accession numbers

The following accessions refer to GenBank (www.ncbi.nlm.nih.gov). Bacterial neomycin phosphotransferase: U55762; cathepsin B: U58000; cathepsin L: Z22767, EU287918, EU287915, AJ279091, EU191984, AJ279093, EU287914, EU195859, DQ534446, EU287917, EU287916, Z22763, DQ533985, Z22764, EF407948, Z22765, EF611824, U62289, Z22769, AJ279092, AY029229, AB009306, AY519972, AY573569, L33771, AF490984, AY277628, U62288, AY519971, DQ533986, Z22766, L33772, AF271385; fatty acid binding protein: AJ250098; μ-class glutathione transferase: M77682, M93434, M77680, M77681, M77679; ω-class glutathione transferase: JX157880; σ-class glutathione transferase: DQ974116; glyceraldehyde phosphate dehydrogenase: AY005475; leucine aminopeptidase: AY644459.

## Results

### *In vitro* maintenance of NEJs

**Figure 1:**
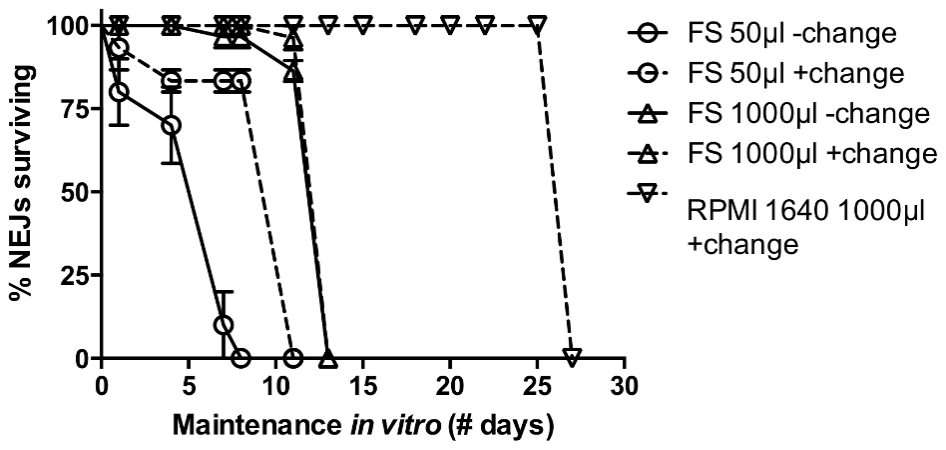
Survival of *Fasciola hepatica* newly excysted juveniles (NEJs) in *Fasciola* saline (FS) vs RPMI 1640. NEJ viability in FS is improved both by regular media changes (−change = without media changes; +change = with media changes), or by maintenance in larger volumes (50 µl vs 1000 µl). Experiments were replicated 3 times, employing 15 worms per treatment. Symbols represent mean±SEM.

Although our early experiments were performed using *Fasciola* saline (FS) maintenance media as described previously [8], we found the viability (assessed as a visual measure of worm motility and morphology; non-motile worms with a visually disrupted tegument were considered dead) of NEJs to be poor over maintenance periods of more than a few days under these conditions (Figure 1). Initial soaks maintained 20 worms in 50 µl FS, in which NEJs survived for no longer than 8 days (50% survival = 5 days). With regular media changes (every 2 to 3 days), maintenance could be extended to a maximum of 11 days (50% survival = 9 days). Maintaining NEJs in a larger volume (1000 µl) improved survival further to a maximum of 13 days (50% survival = 12 days), although in this case NEJ survival was not improved by regular media changes. In order to track protein dynamics occurring on timescales beyond 13 days, we employed RPMI 1640 media, finding that this media supported NEJ survival for up to 27 days when maintained in aseptic conditions under a 5% CO_2_ atmosphere. All of the data presented below were derived from experiments employing RPMI 1640, although comparative experiments performed in both media across multiple targets over 72 h timescales illustrated no significant difference in RNAi efficacy between FS and RPMI 1640.

### RNAi transcript knockdown dynamics

This study aimed primarily to identify a set of optimised RNAi experimental conditions that would permit simple and robust gene knockdown in a widely used and commercially available strain of *F. hepatica*. We describe a set of simple, soaking-based methods that permit rapid, robust and persistent knockdown of target transcripts, and address target-specific aspects of the timecourse of this RNAi response, which is particularly evident in the time lag between transcript and protein knockdown.

### Constant exposure to trigger

**Figure 2:**
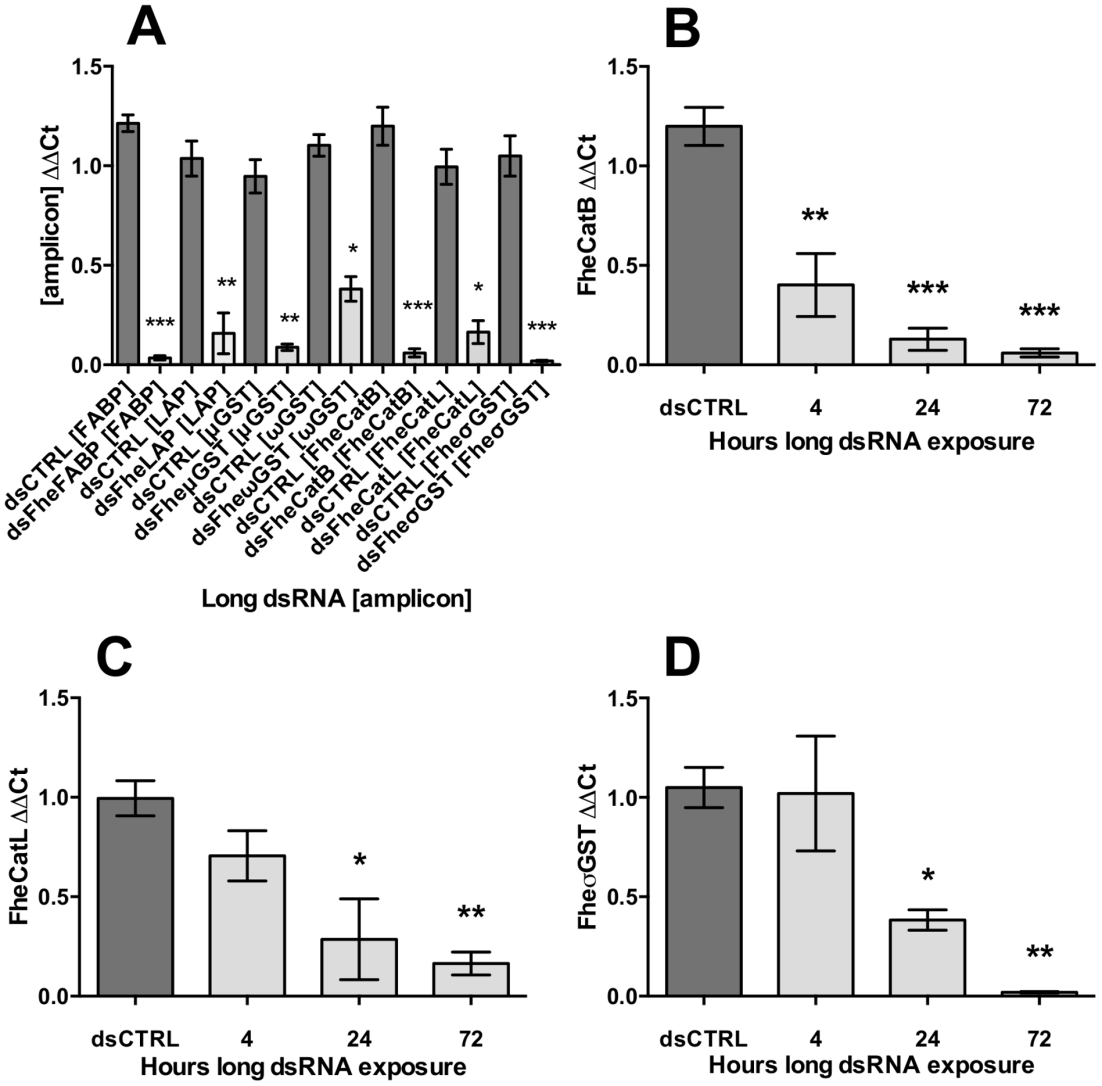
Time-dependent transcript knockdown under constant exposure to long double-stranded (ds)RNA. A, knockdown of virulence gene target transcripts after 72 h exposure to long dsRNA (CTRL, negative control; FABP, fatty acid binding protein; LAP, leucine aminopeptidase; μGST, μ-class glutathione transferase; ωGST, ω-class glutathione transferase; CatB, cathepsin B; CatL, cathepsin L; σGST, σ class glutathione transferase); B–D, time-dependent knockdown of cathepsin B (B), cathepsin L (C), σ-class glutathione transferase (D). Square brackets e.g. [CatB] represent qPCR amplicon. Target ΔΔCt (Y-axes) represents ratio of abundance of target transcript to a reference gene (glyceraldehyde phosphate dehydrogenase, GAPDH), in treated samples, relative to the abundance of those transcripts in untreated samples. Statistical significances are indicated relative to effects of negative control dsRNA (dsCTRL, complementary to neomycin phosphotransferase). dsCTRL treatments were performed in parallel with all experimental treatments. Experiments were repeated ≥4 times, employing 20–30 flukes per replicate. *, P<0.05; **, P<0.01; ***, P<0.001. Symbols represent mean±SEM.

Initial experiments maintained NEJs under constant exposure to long dsRNA for periods up to 72 h, using the same dsRNA molecules/sequences, concentrations, and exposure conditions previously reported [8]. These conditions did not, in our hands, trigger aberrant motility phenotypes. Upon analysing transcriptional knockdown dynamics from these soaks, we established that there was no detectable difference in transcript knockdown level between 50 ng/µl and 100 ng/µl dsRNA exposures. All subsequent experiments employed 50 ng/µl dsRNA, unless stated otherwise. Although we were unable to detect the reduction in FheCatL transcript reported by McGonigle *et al.* after 4 h exposure, simply extending the length of dsRNA exposure did elicit time-dependent suppression of CatL, as well as our other target transcripts after 72 h (FheFABP: 0.03±0.01, P<0.001, *n* = 3; FheLAP: 0.16±0.10, P<0.01, *n* = 3; FheμGST: 0.09±0.02, P<0.01, *n* = 3; FheωGST: 0.38±0.06, P<0.05, *n* = 3; Figure 2A). Note that these four targets were assayed only by qPCR at the 72 h timepoint. Intermediate time-course exposures, performed to 4, 24 and 72 h (Figure 2B–D), showed that both FheCatL (24 h: 0.29±0.20, P<0.05; *n* = 4; 72 h: 0.16±0.06, P<0.01, *n* = 5), and FheσGST (24 h: 0.38±0.05, P<0.05 n = 5; 72 h: 0.02±0.004, P<0.01, *n* = 4) exhibited significant transcript knockdown from 24 h onwards, while FheCatB displayed a uniquely rapid response, with transcript knockdown detectable after only 4 h exposure to long dsRNA (4 h: 0.40±0.16, P<0.05, *n* = 6; 24 h: 0.13±0.06, P<0.05, *n* = 4; 72 h: 0.06±0.02, P<0.05, *n* = 4). None of these experiments resulted in detectable aberrant phenotypes. The major drawback of this approach was that NEJs maintained under constant exposure to long dsRNA in such small volumes exhibited rapidly declining viability beyond 2–3 days *in vitro* (Figure 1), which occluded our detection of potential impacts of RNAi on worm behaviour.

### Burst exposure to long dsRNA trigger

**Figure 3:**
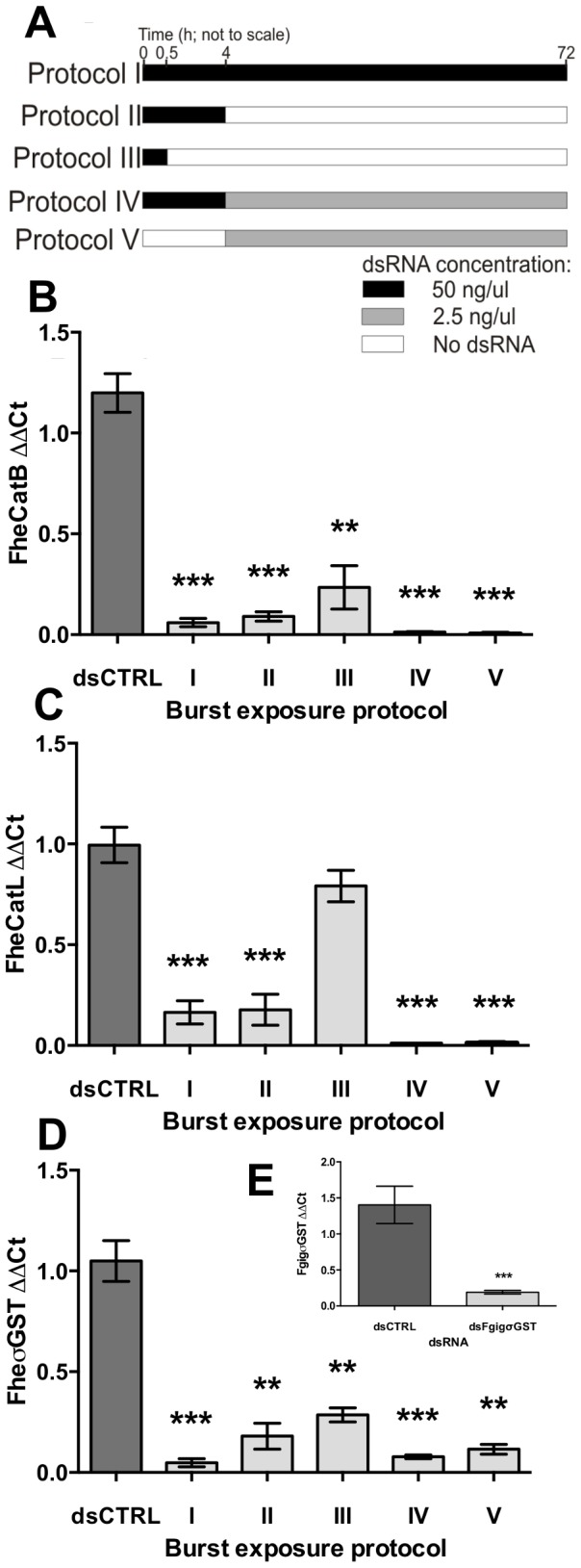
Transcript knockdown following burst exposure to long double-stranded (ds)RNA. Exposure conditions are indicated in panel A, where newly-excysted juveniles were incubated in 50 ng/µl dsRNA (black bar), 2.5 ng/µl dsRNA (grey bar) or zero/sub-effective dsRNA (clear bar), for time periods as indicated in scale. Media were replaced at 2–3 day intervals for the duration of the experiments. Transcript knockdown was assayed in all cases by quantitative PCR, 72 h after start of soak. Target ΔΔCt (Y-axes) represents ratio of abundance of target transcript (B, cathepsin B; C, cathepsin L; D, sigma-class glutathione transferase) to a reference gene (glyceraldehyde phosphate dehydrogenase, GAPDH) in treated samples, relative to the abundance of those transcripts in untreated samples. E, Silencing of σGST ortholog in *Fasciola gigantica*, using long dsRNA exposure under Protocol IV conditions. Data expressed as mean±SEM. Statistical significances are indicated relative to effects of negative control dsRNA (dsCTRL, complementary to neomycin phosphotransferase). dsCTRL treatments were performed in parallel with all experimental treatments. Experiments were repeated ≥3 times, employing 20–30 flukes per replicate. *, P<0.05; **, P<0.01; ***, P<0.001. Symbols represent mean±SEM.

Poor survival of NEJs under the constant exposure conditions described above (here and in Figure 3 referred to as Protocol I) was mitigated by either regular changes (every 2–3 days), and/or maintenance in an increased volume (1 ml) of media. Since such approaches would require prohibitively large amounts of RNAi trigger during longer maintenance periods, we investigated the efficacy of short, low volume “burst” exposures to long dsRNA, followed by longer-term incubation in the absence of dsRNA under optimal maintenance conditions. These experiments were performed over a 72 h timecourse (Figure 3). Initial experiments (Protocol II) incorporated a 4 h dsRNA burst exposure, after which dsRNA was removed (or reduced to irrelevant levels) by a series of six washes in 1 ml RPMI 1640, before maintenance for 68 h in 1 ml RPMI 1640. In all cases, target transcript knockdowns had developed to highly significant levels by the end of this maintenance period (FheCatB: 0.09±0.02, P<0.001, *n* = 4; FheCatL: 0.18±0.08, P<0.001, *n* = 5; FheσGST: 0.18±0.06, P<0.001, *n* = 4; Figure 3B–D). Transcript knockdown was triggered even when the burst exposure period was reduced to as little as 30 min (Protocol III), although this knockdown was statistically significant only in the cases of FheCatB and FheσGST (FheCatB: 0.23±0.11, P<0.01, *n* = 3; FheCatL: 0.79±0.08, P>0.05, *n* = 3; FheσGST, 0.29±0.03, P<0.01, *n* = 5). A 15 min burst exposure was ineffective. Since NEJs were effectively removed from dsRNA following the period of burst exposure (and in the cases of FheCatL and FheσGST, knockdown had not been present after 4 h, Figure 2C,D), these findings indicate that dsRNA taken up by NEJs during their burst exposure period was sufficient to trigger the development of knockdown during subsequent incubation in the absence of exogenous dsRNA. In two out of three cases, the highest levels of knockdown were achieved over 72 h using the less onerous method (Protocol IV) of simply adding 1 ml RPMI 1640 for 68 h after the 4 h soak period (i.e. dilution of dsRNA to approximately 2.5 ng/µl) (FheCatB: 0.02±0.003, P<0.001, *n* = 3; FheCatL: 0.01±0.0003, P<0.001, *n* = 5; FheσGST: 0.08±0.01, P<0.001, *n* = 7). To ascertain the importance of the initial high-concentration burst exposure to this response, we also performed soaks in the absence of this step (Protocol V), simply soaking NEJs for 68 h in 2.5 ng/µl long dsRNA. This approach also delivered highly significant levels of transcript knockdown (FheCatB: 0.01±0.003, P<0.001, *n* = 6; FheCatL: 0.01±0.003, P<0.001, *n* = 7; FheσGST: 0.12±0.02, P<0.01, *n* = 3), indicating that the concentration threshold of the NEJ long dsRNA uptake mechanism is ≤2.5 ng/µl. For reasons of efficacy and convenience, all subsequent experiments in this study employed Protocol IV.

### RNAi in *Fasciola gigantica*

In order to establish the applicability of these RNAi methods to the tropical fluke, *F. gigantica*, we applied long dsRNA complementary to FgigσGST to *F. gigantica* NEJs using Protocol IV methods. This triggered effective knockdown of FgigσGST transcript (0.19±0.03, P<0.001, *n* = 5; Figure 3E). These data represent the first demonstration of RNAi in *F. gigantica*. Note that we did not examine suppression of FgigσGST protein, nor any of the other genes referred to in this study, in *F. gigantica*.

### Persistent transcript knockdown leads to suppression of target protein

**Figure 4:**
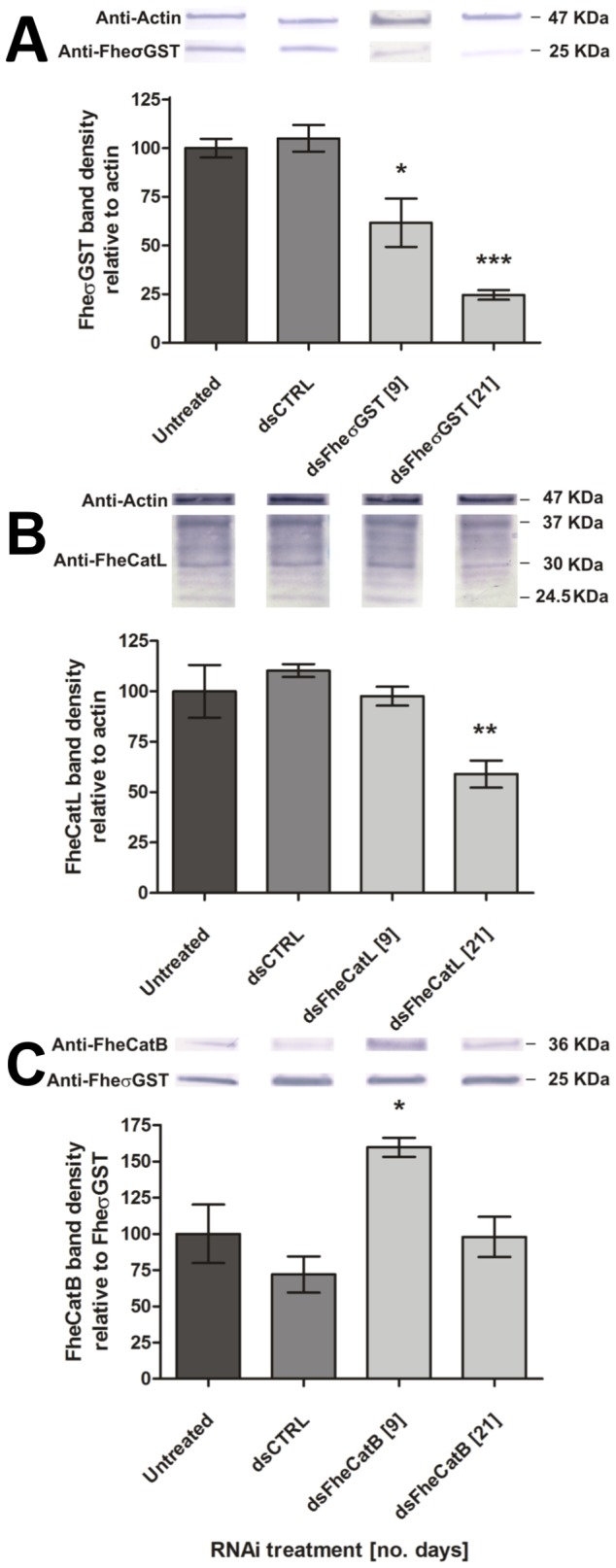
Effects of RNA interference on target protein abundance during maintenance *in vitro*. Densitometry analysis performed of bands generated by immunoblot analyses of newly-excysted juvenile (NEJ) crude protein extracts, at 9 and 21 days post exposure to long double-stranded (ds)RNA: A, σGST; B, cathepsin L, C, cathepsin B. Each panel includes typical examples of target bands imaged from immunoblots using: (A) sigma glutathione transferase antiserum (25 KDa), and actin antiserum (47 KDa) loading control; (B) cathepsin L antiserum (preproenzyme, 37 KDa; partially-processed intermediate, 30 KDa; mature enzyme, 24.5 KDa), and actin antiserum (47 KDa) loading control; (C) anti-cathepsin B antiserum (preproenzyme, 36 KDa), and anti-FheσGST (sigma glutathione transferase, 25 KDa) loading control. Bar graphs illustrate densitometric analyses of western blot protein bands, where target band density is normalised to loading control band density, and expressed relative to untreated sample (where untreated control = 100%). Statistical significances are indicated relative to effects of negative control dsRNA (dsCTRL, complementary to neomycin phosphotransferase). Untreated and dsCTRL-treated samples were performed in parallel with all experimental treatments; these columns represent means of both time points. Experiments were repeated ≥3 times, employing 50–60 flukes per replicate. *, P<0.05; **, P<0.01; ***, P<0.001. Symbols represent mean±SEM.

Triggering highly significant levels of transcript knockdown over the 72 h periods described above did not impact on the survival or behaviour of NEJs maintained *in vitro*. Supporting this observation, western blot analyses of FheCatB, FheCatL and FheσGST protein performed at 18 h and 72 h confirmed that target protein levels had not changed significantly at these timepoints. Extension of the *in vitro* maintenance period enabled detection of FheσGST protein suppression after 9 days (61.7±12.43%, p<0.05, *n* = 4; Figure 4A), which had increased further by 21 days (24.60±2.49%, P<0.001, *n* = 3; Figure 4A). Suppression of FheCatL protein was not detectable until 21 days (58.97±6.74, P<0.01, *n* = 3; Figure 4B). FheCatB protein levels were not reduced vs controls at all time points up to and including 21 days (Figure 4C).

**Figure 5:**
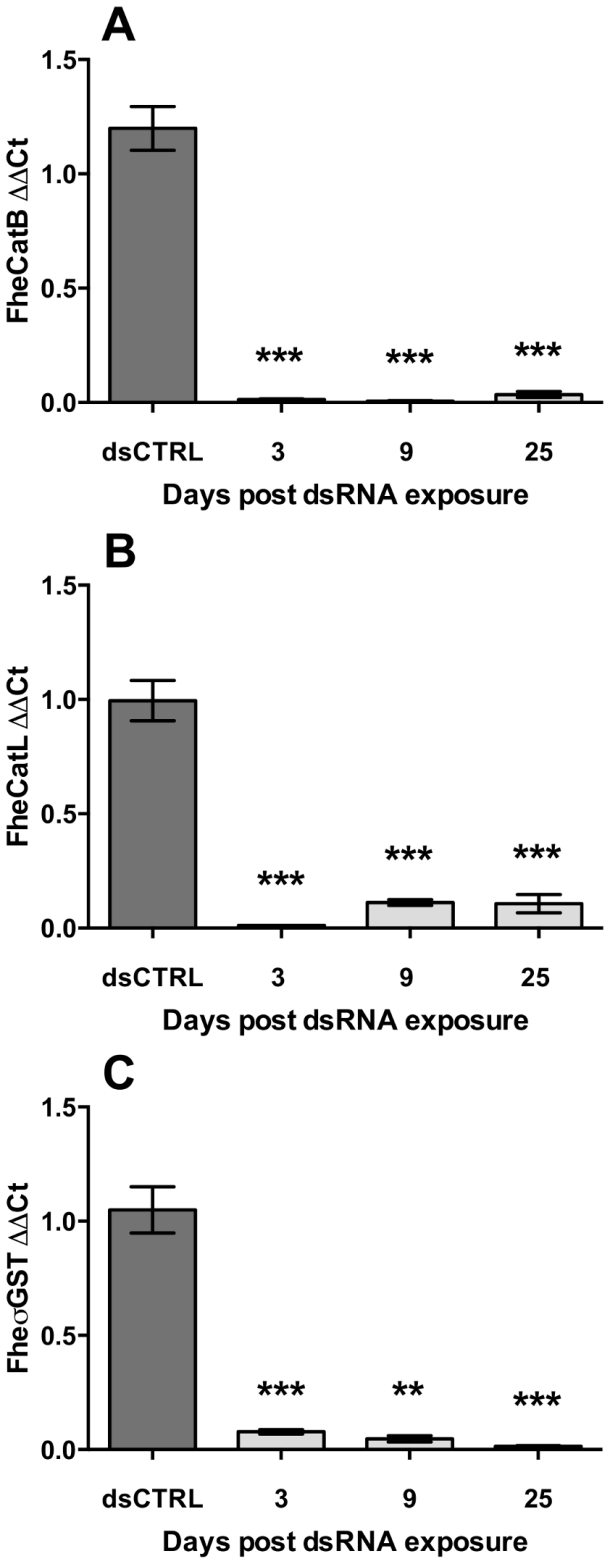
Persistence of transcript knockdown during maintenance *in vitro*. Following exposure to target long double-stranded (ds)RNA (A, cathepsin B; B, cathepsin L; C, sigma-class glutathione transferase), newly-excysted juveniles (NEJs) were maintained *in vitro* for up to 25 days. NEJs were collected and assayed by quantitative PCR at 3, 9 and 25 days post dsRNA exposure. Target ΔΔCt (Y-axes) represents ratio of abundance of target transcript to a reference gene (glyceraldehyde phosphate dehydrogenase, GAPDH), in treated samples, relative to the abundance of those transcripts in untreated samples. Statistical significances are indicated relative to effects of negative control dsRNA (dsCTRL, complementary to neomycin phosphotransferase). dsCTRL treatments were performed in parallel with all experimental treatments. Experiments were repeated ≥3 times, employing 20–30 flukes per replicate. *, P<0.05; **, P<0.01; ***, P<0.001. Symbols represent mean±SEM.

PCR analyses showed persistence of highly significant transcript knockdown for all three targets throughout a 25 day period of maintenance *in vitro* (day 25 FheCatB: 0.03±0.01, P<0.001, *n* = 5; FheCatL: 0.12±0.04, P<0.001, *n* = 5; FheσGST: 0.01±0.003, P<0.001, *n* = 4; Figure 5), suggesting that inadequate target transcript knockdown is probably not responsible for the absence of detectable FheCatB protein suppression at later time points. Notably, even in experiments where suppression of FheCatL or FheσGST protein was detectable, over maintenance periods up to 25 days, we observed no changes in NEJ survival or behaviour compared to time-matched negative controls.

### Both long dsRNA and siRNA triggers are effective at low ng/µl concentrations, and display concentration-dependent impacts on target transcript abundance

**Figure 6:**
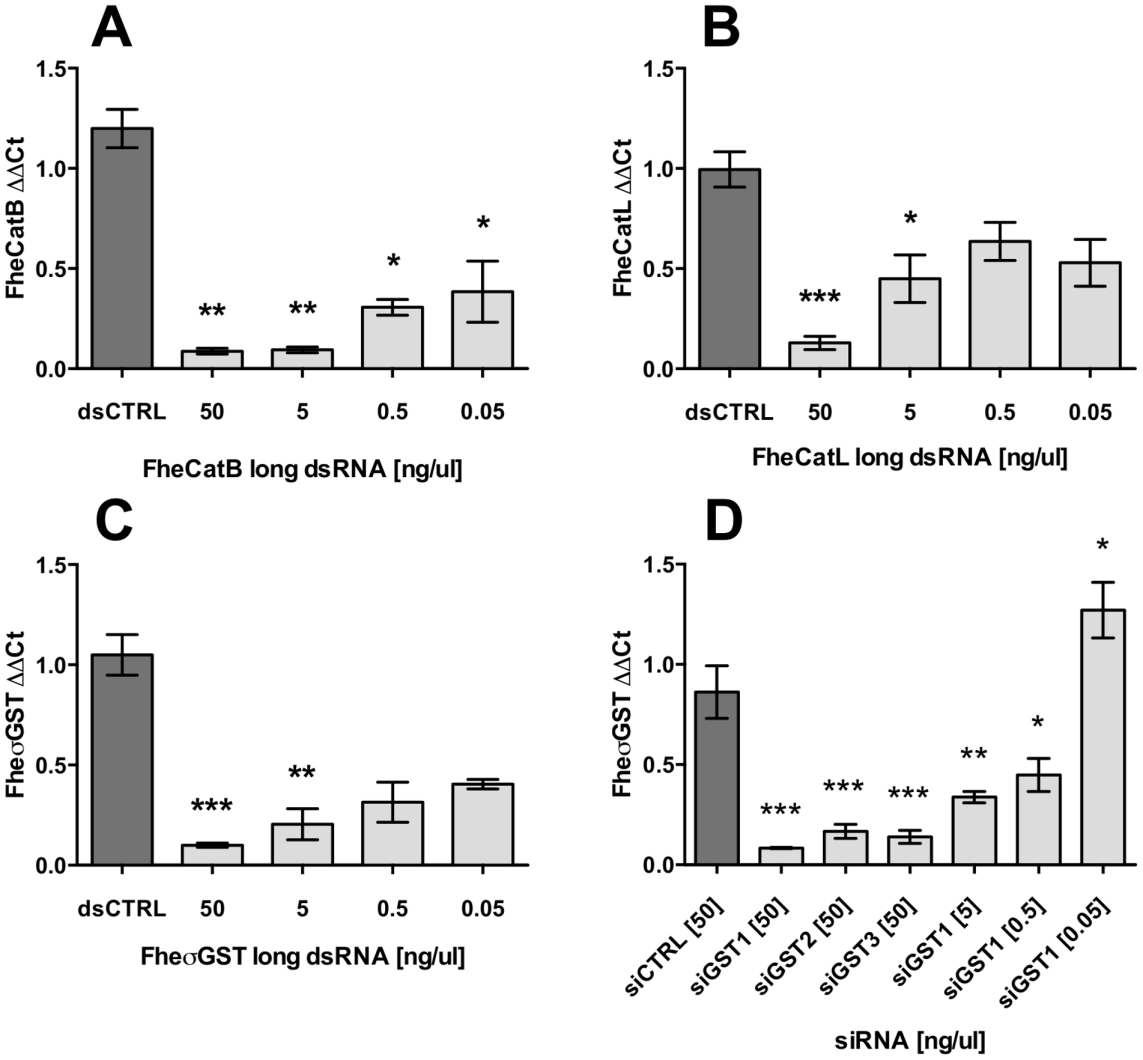
Concentration-dependent effects of long double-stranded (ds)RNA and short interfering (si)RNA triggers on transcript knockdown. Target long dsRNAs (A, cathepsin B; B, cathepsin L; C, sigma-class glutathione transferase), and σGST siRNAs (D) serially diluted in RPMI 1640, were applied to newly-excysted juveniles at the concentrations indicated. Transcript knockdown was assayed in all cases by quantitative PCR, 72 h after start of soak. Target ΔΔCt (Y-axes) represents ratio of abundance of target transcript to a reference gene (glyceraldehyde phosphate dehydrogenase, GAPDH), in treated samples, relative to the abundance of those transcripts in untreated samples. Statistical significances are indicated relative to effects of negative control dsRNA (dsCTRL, complementary to neomycin phosphotransferase), or siRNA (siCTRL, commercially supplied scrambled sequence). Control treatments were performed in parallel with all experimental treatments. dsCTRL and siCTRL bars illustrate the impacts of the highest trigger concentrations tested (50 ng/ul). Experiments were repeated ≥3 times, employing 20–30 flukes per replicate. *, P<0.05; **, P<0.01; ***, P<0.001. Symbols represent mean±SEM.

**Figure 7:**
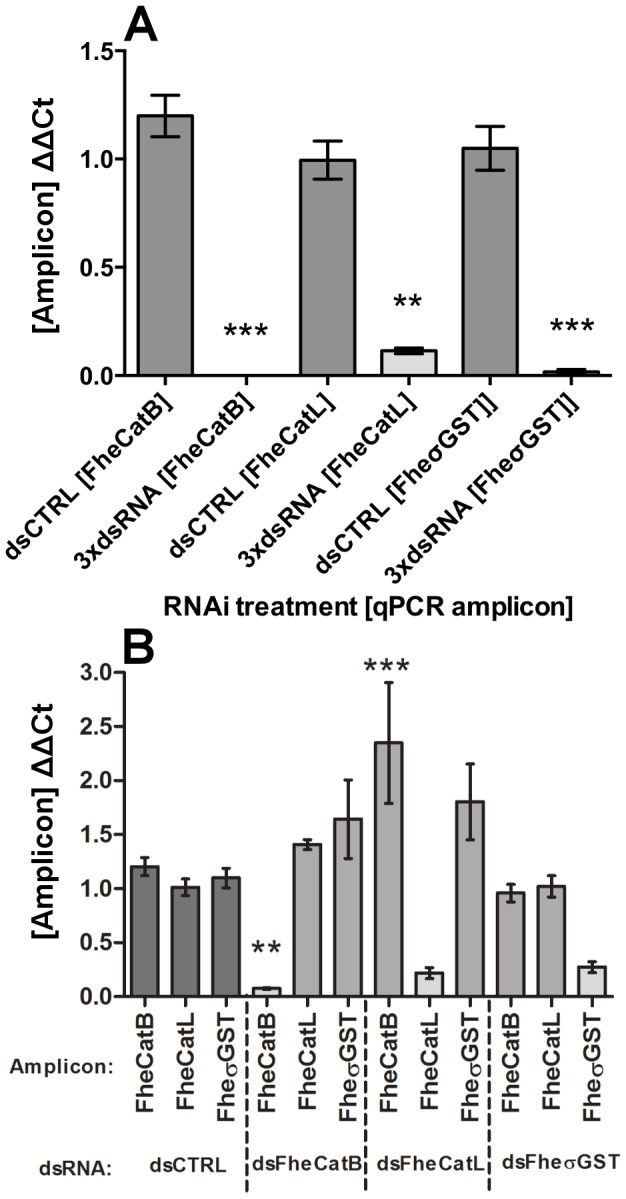
Combinatorial silencing and specificity of cathepsin B, cathepsin L and sigma glutathione transferase RNAi on target transcripts. (A) Newly-excysted juveniles exposed to a cocktail of long double-stranded (ds)RNA against all three targets (individual dsRNAs at 16.7 ng/µl concentration, final total dsRNA concentration, 50 ng/µl). (B) RNAi specificity, assayed via non-specific transcriptional impacts of three target long dsRNAs. Target ΔΔCt (Y-axes) represents ratio of abundance of target transcript to a reference gene (glyceraldehyde phosphate dehydrogenase, GAPDH), in treated samples, relative to the abundance of those transcripts in untreated samples. Statistical significances are indicated relative to effects of negative control dsRNA (dsCTRL, complementary to neomycin phosphotransferase). dsCTRL treatments were performed in parallel with all experimental treatments. Experiments were repeated 4 times, employing 20–30 flukes per replicate. *, P<0.05; **, P<0.01; ***, P<0.001. Symbols represent mean±SEM.

All long dsRNAs tested triggered reproducible, concentration-dependent knockdown of target transcripts. Although our standard soak protocol employed 50 ng/µl long dsRNA, effective transcript knockdown was also achievable using concentrations an order of magnitude lower (5 ng/µl: FheCatB, 0.09±0.01, P<0.01, *n* = 3; FheCatL, 0.45±0.12, P<0.05, *n* = 5; FheσGST, 0.20±0.08, P<0.01, *n* = 4; Figure 6A–C). Statistically-significant transcript suppression relative to control treatments was apparent with as little as 0.05 ng/µl long dsRNA in the case of FheCatB (0.38±0.15, P<0.05, *n* = 3; Figure 6A). These observations are of practical importance in permitting the possibility of combinatorial RNAi (i.e. the silencing of multiple targets in parallel), an approach that was demonstrated here by combining FheCatB, FheCatL and FheσGST long dsRNAs to a final total concentration of 50 ng/µl ( = each individual dsRNA at 16.7 ng/µl). Under Protocol IV conditions, this method triggered knockdown of all three targets in parallel to a level not significantly different from that achieved in individual soaks (Figure 7A). No phenotypic impacts were observed during the 72 h combinatorial RNAi timecourse, although we did not investigate impacts on protein suppression during these experiments.

In order to establish the efficacy of siRNAs in comparison to long dsRNA, we employed three individual siRNAs complementary to FheσGST (siGST1–3). All three siRNAs triggered highly significant knockdown of FheσGST transcript at 50 ng/µl (siGST1: 0.08±0.003, P<0.0001, *n* = 3; siGST2: 0.17±0.04, P<0.001, *n* = 3; siGST3: 0.14±0.03, P<0.001, *n* = 3; Figure 6D). Titration of the most potent siRNA, siGST1, showed concentration-dependent effects that were significant at a concentration 10-fold more potent than long dsRNA (siGST1 5 ng/µl: 0.34±0.03, P<0.01, *n* = 5; 0.5 ng/µl: 0.45±0.08, P<0.05, *n* = 5; Figure 6D).

### Long dsRNA transcript knockdown specificity

RNAi off-target impacts were assessed by amplifying non-target amplicons from long dsRNA-treated cDNAs (Figure 7B). While these analyses showed no evidence for off-target knockdown by any of our long dsRNAs, we did observe some up-regulations of transcripts following treatment with non-target long dsRNAs. This phenomenon was only statistically-significant in the case of the impact of FheCatL dsRNA on FheCatB. Further experimentation is needed to fully characterise the consequences, and biological significance, of such up-regulation on target protein levels.

## Discussion

Development of RNAi-based gene silencing methods is pivotal for the effective exploitation of burgeoning database resources for parasitic helminths. *Schistosoma* spp. trematodes provide arguably the best example of how the effective confluence of genomic resources with powerful RNAi protocols can promote the systems-level understanding of helminth parasite biology [25]. *Fasciola* spp. liver fluke are increasingly the focus for genome and transcriptome sequencing projects [9]–[12], but have not seen the widespread adoption of RNAi and related tools by the liver fluke research community. As a means of promoting such uptake, this study aimed to develop a standardised set of RNAi protocols applicable to NEJs of a widely used *F. hepatica* strain. We have shown that transcriptional knockdown can be triggered in seven virulence gene targets, simply by soaking NEJs in a solution of tissue culture media containing long dsRNA, or siRNA, trigger molecules. In three targets subject to in-depth optimisation of variables, transcript knockdown occurred in a concentration-dependent manner that was invariably rapid and persistent. Although target-specific RNAi dynamics were apparent in terms of the rapidity of transcriptional response to dsRNA trigger, these dynamics manifested most notably in the length of the lag phase between transcript knockdown and protein suppression. This lag phase was appreciable in all cases, with FheσGST protein suppression developing only 9 days following exposure to long dsRNA, FheCatL suppression was not evident until 21 days post-exposure. We were unable to detect FheCatB suppression at any point up to and including 21 days. These manipulations did not trigger any measurable changes in NEJ viability or behaviour *in vitro*. Nevertheless, the protocols described here represent a relatively simple means to achieve targeted transcript and protein knockdowns in the absence of advanced manipulations such as electroporation [7], [26], biolistics [27], transfection reagents [28], or genetic transformation [29], such as have been described in other trematodes. Alongside development of appropriate functional assays, these RNAi protocols should enable gene function analysis even by laboratories with limited molecular expertise, and may lend themselves to multiple-throughput screening approaches to target validation.

Despite the successful knockdowns reported here, our data contrast with those of a previous study [8], which described extremely rapid RNAi dynamics in *F. hepatica* NEJs of both Oberon and Fairhurst isolates (see [30] for discussion of fluke isolates). In the case of cathepsins B and L, McGonigle *et al.* [8] demonstrated suppression of both target transcript and protein after just 4 h exposure to long dsRNA, correlating at this time-point with reduced migration through in an *in-vitro* rat gut penetration assay. Although we attempted initially to replicate the molecular aspects of those experiments using quantitative methods, we employed a commercially available and widely used strain of *F. hepatica*, the US Pacific North-West Wild strain, instead of Oberon and Fairhurst isolates. After 4 h exposure to long dsRNA, we observed reliable knockdown only of FheCatB transcript (Figure 2B). FheCatL remained refractory to transcript knockdown after 4 h exposure, even over multiple replicates performed over several days using independently prepared batches of long dsRNA. Furthermore, throughout all of the experiments described here, we never observed any of the “erratic locomotion and paralysis” phenotypes reported by McGonigle *et al.* Even in soaks employing 1 µg/µl FheCatL dsRNA (10 fold higher than that used by McGonigle *et al.*), US Pacific North West strain NEJs displayed no visibly different behaviour from controls, and no FheCatL transcript knockdown, over a 4 h exposure period. After addressing the relevant experimental variables between our studies, the primary difference that remained was our use of different *F. hepatica* isolates. Variable RNAi susceptibility has been observed between strains of the nematode, *Caenorhabditis elegans* [31], where inter-strain differences in somatic RNAi competency correlate with differences in the capacity to mount an anti-viral response. A similar situation could conceivably exist between genetically-distinct liver fluke isolates, related to sequence-level differences in RNAi pathway proteins, although we have been unable to test this hypothesis directly in *F. hepatica* Oberon and Fairhurst isolates. However, planned draft genome sequences from flukes of diverse genetic backgrounds, and anthelmintic susceptibilities (Steve Paterson, Jane Hodgkinson, personal communication) may shed further light on potential isolate-related differences in RNAi mechanics. While strain/isolate-specific RNAi susceptibilities could explain differences in transcriptional responses to RNAi triggers as discussed here, based on our current knowledge of RNAi mechanisms in other organisms they do not adequately explain the differences in rates of protein suppression observed between this work and that of McGonigle et al. [8]. One may speculate that the proteins in question may exhibit different half-lives in the distinct isolates used in the respective studies, i.e. different post-RNAi protein suppression rates could be due to longer target protein half-life in the Pacific North West strain than in the Oberon/Fairhurst isolates.

The transcript knockdown data described here clearly illustrate that RNAi can be triggered using very simple methodology in NEJs of the US Pacific North West Wild strain of *F. hepatica*. Burst exposure experiments (Figure 3), and titrations of long dsRNAs and siRNAs (Figure 6), show that even limited temporal exposure to (30 min) or low concentrations (0.05 ng/µl) of long dsRNA are sufficient to trigger the development of transcript knockdown over a 72 h period. Transcript knockdowns also persist at highly significant levels over maintenance periods of at least 25 days – this is reminiscent of *S. mansoni*, where cathepsin B knockdown was reportedly sustained for at least 40 days following delivery of long dsRNA by electroporation [19]. The transcriptional acquiescence of NEJs to exogenously triggered RNAi suggests the presence of a suite of efficient cellular RNAi mechanisms, presumably involving a rapid and efficient dsRNA uptake mechanism. This mechanism may involve SID (Systemic RNAi Defective) homologues, dsRNA-gated channels responsible for transmembrane-transport of dsRNA molecules, identified originally in *C. elegans* [32]–[34] with orthologues described in *S. mansoni* and several other disparate genera [35]–[41]. The development of transcript knockdown over several days following exposure to dsRNA suggests a slow intercellular spread of dsRNA trigger from point of entry, and/or the presence of an RNA-dependent RNA polymerase (RdRP)-like secondary siRNA-based amplification system (although RdRPs have not yet been described in flatworms, our unpublished bioinformatics analyses do suggest the presence of RdRP-like sequences in available fluke datasets).

Throughout this study, no impacts were observed on NEJ viability or behaviour during RNAi experiments. These observations were supported in early experiments by western blots performed at 18 h and 72 h, which revealed no changes in levels of FheCatB, FheCatL or FheσGST protein. Given that some schistosome genes require maintenance periods of up to 14 days post-electroporation before evidence of protein suppression or phenotype [42], we hypothesised that protein suppression could be similarly achieved in our targets simply by maintaining worms for longer *in vitro* following dsRNA exposure, in order to permit run-down of protein levels. Under such extended maintenance periods, a western blot-based approach detected FheσGST suppression at 9 and 21 days post-exposure, while FheCatL suppression was not detectable until 21 days post-exposure. No suppression of FheCatB protein was detected at any point during the 21-day timecourse, although an apparent upregulation of cathepsin B was detected in our day 9 samples. Although we might assume that these differential dynamics simply reflect the dissimilar run-down rates of functionally distinct proteins, differences in the sequence diversity of these targets might equally impact on our ability to detect changes in protein abundance: FheσGST is a single copy gene for which our long dsRNA had very little sequence identity to other transcripts, and for which we had access to a specific FheσGST-antiserum. In this case, detection of protein suppression is a relatively simple matter, where changes in immunoblot band density can be expected to provide an accurate read-out for changes in protein abundance. In the case of multi-gene cathepsin families, the ability to trigger and detect promiscuous changes in protein abundance may depend on the relative specificity/cross-reactivity of dsRNA and antisera, both within and between members of the target gene family. In the case of cathepsin L, where 78% mean sequence similarity exists between the seven recognised *F. hepatica* cathepsin L clades, including many regions of identity of >20 nt (consistent with generation of siRNAs by Dicer), long dsRNA targeted to any clade is likely to trigger promiscuous knockdown of the broader gene family. Polyclonal antisera are likely to be similarly promiscuous across the multiple cathepsin L protein clades, hence our detection of FheCatL protein suppression by 21 days post dsRNA exposure. In the case of cathepsin B however, sequence similarity across the ten recognised clades is somewhat lower (mean 53%), and our long dsRNA (in this study, complementary to FheCatB2) does not possess significant stretches of identity with the other clades, reducing the likelihood of promiscuous, broad knockdown of the FheCatB gene family. In contrast, the cathepsin B polyclonal antiserum used here is likely to cross-react with several cathepsin B clades. Under these circumstances, suppression of FheCatB2 alone may not have been detected by our immunoblot methodology unless it accounted for the major proportion of expressed cathepsin protein in the tissue extract (which is unlikely [43]). This issue could be addressed quite simply in future studies by employing a cocktail of dsRNAs representing all of the FheCatB clades expressed in NEJs [44]. The combinatorial RNAi data presented in Figure 7A, showed that at least three targets could be silenced in parallel, supporting the feasibility of such an approach. An alternative to the immunodetection approaches used here is 2D electrophoresis-based sub-proteomics, which has shown promise in detecting RNAi-induced suppression of individual proteins in multi-gene families in other systems (LaCourse et al., 2008 [45]). Another technical consideration is that even where FheCatL protein suppression was detected, our blots showed that it was most apparent in the bands representing the 24.5 KDa mature enzyme and 30 KDa processed intermediate, with little reduction in band density apparent for the 37 KDa preproenzyme (Figure 4). This phenomenon is likely due to the storage of cathepsin L preproproteins as inactive zymogens within secretory vesicles (a location likely to limit proteolytic breakdown), prior to exocytotic release [43], [46]–[49]). This may also have inhibited detection of FheCatB protein suppression, since only the preproprotein form was detected by our cathepsin B antiserum. Alternatively, the apparently long protein half-lives seen here in the face of transcript ablation may simply reflect our inability to provide adequate maintenance conditions for obligate intra-mammalian parasites, which has resulted in abnormal cell division and cellular protein dynamics that artificially inhibit protein turnover. Given the high and sustained levels of transcript knockdown reported here, it seems unlikely that limiting transcript knockdown is responsible for the apparent lack of protein suppression in our experiments. Nevertheless, it is possible that the delivery of dsRNA by other methods such as electroporation, biolistics, transfection reagents, or genetic transformation [18], [19], [27]–[29] may increase transcript knockdown levels, hastening the validity of phenotypic measurements. Varying the mechanism of dsRNA delivery is a factor that could be addressed by future experiments.

Despite detectable suppression of two out of three target proteins, we observed no impact of RNAi on NEJ behaviour or viability. Although these observations contrast with previous reports of paralysis and compromised motility following RNAi of cathepsin B and L in liver fluke [8], they are consistent with silencing of these virulence genes in other organisms. Where RNAi has been used to target cathepsin B, cathepsin L or GSTs in multicellular eukaryotes in an *in vitro* setting, phenotypic consequences have been reported only under functionally-defined assay settings such as feeding/growth [26], [29], [50], [51], drug susceptibility [52], [53], or in dynamic systems measuring reproductive output or molting [54]–[57]. Under settings where organisms are not subject to functional assay but merely observed in the presence of RNAi (such as the present study), no impacts on survival or motility have been reported following RNAi of cathepsin B, cathepsin L, or GSTs [20], [58]–[62]. The lack of impact on survival/viability following RNAi *in vitro* is not necessarily a poor reflection on the therapeutic potential of these targets, merely a confirmation of the complexity and subtlety of the host-parasite interface that highlights the need for adequately sensitive assay systems. *In vivo* assays, where RNAi impacts on worm virulence are assessed via the ability of an RNAi-treated parasite to develop to patency within a host organism, have been employed in helminths [25], [54], [63]–[66]. Although *in vitro* RNAi results cannot always be recapitulated *in vivo* [64], the persistent knockdowns achieved in the current study suggest the amenability of *F. hepatica* NEJ RNAi studies to such an *in-vivo* infection assay. Efforts to develop *in vivo*, and tailored *in vitro* assays for several targets are currently ongoing in our laboratories. These assays will permit the development of RNAi to its full potential in liver fluke.

This study has shown that RNAi protocols, based simply on soaking in long dsRNA or siRNA even for brief periods, are capable of triggering robust, persistent transcript knockdown in liver fluke NEJs. However, notes of caution are offered in terms of the possibility of liver fluke isolate-specific differences in RNAi mechanics, and evidence for variable lag periods between transcript and protein suppression; the latter highlight the need for careful assessment of target dynamics prior to the application and interpretation of phenotypic assays. The simple methods described here should be widely applicable within the liver fluke research community, and should bolster efforts both to investigate liver fluke gene function and identify novel targets for therapy.

## Supporting Information

Figure S1 **PCR primer design strategy for induction and detection of RNA interference (RNAi) in** ***Fasciola hepatica*** **cathepsin L sequences.** A, schematic layout of PCR primers used for generation of double stranded (ds)RNA templates labelled with T7 RNA polymerase promoter sequences, and a quantitative (q)PCR amplicon. Note that both ‘sense’ and ‘antisense’ dsRNA templates are generated, from which sense and antisense RNA strands are generated respectively, before being annealed to generate dsRNA. B, nucleotide sequence alignment of available *Fasciola hepatica* cathepsin L sequences, showing positioning of primers and their cross-reactivity across clades. Cathepsin L clades are indicated in sequence titles [CL1A, 1B, 2–6] as described elsewhere [67], [68]. This alignment was performed in mid 2010, these sequences represent those available in GenBank at that time, accession numbers refer to GenBank.
(DOCX)

Figure S2 **PCR primer design strategy for induction and detection of RNA interference (RNAi) in** ***Fasciola hepatica*** **μ-class glutathione transferase (GST) sequences.** A, schematic layout of PCR primers used for generation of double stranded (ds)RNA templates labelled with T7 RNA polymerase promoter sequences, and a quantitative (q)PCR amplicon. Note that both ‘sense’ and ‘antisense’ dsRNA templates are generated, from which sense and antisense RNA strands are generated respectively, before being annealed to generate dsRNA. B, nucleotide sequence alignment of available *Fasciola hepatica* μ-GST sequences, showing positioning of primers and their cross-reactivity between sequences. GST titles and accession numbers are indicated. This alignment was performed in mid 2010, these sequences represent those available in GenBank at that time, accession numbers refer to GenBank.
(DOCX)
