# Supplementary material for: RNAi Dynamics in Juvenile Fasciola spp. Liver Flukes Reveals the Persistence of Gene Silencing In Vitro
Source: PLoS Negl Trop Dis. 2014 Sep 25;8(9):e3185. doi: 10.1371/journal.pntd.0003185 (PMC4177864; doi:10.1371/journal.pntd.0003185)
Supplement: Figure S1 — PCR primer design strategy for induction and detection of RNA interference (RNAi) in Fasciola hepatica cathepsin L sequences. A, schematic layout of PCR primers used for generation of double stranded (ds)RNA templates labelled with T7 RNA polymerase promoter sequences, and a quantitative (q)PCR amplicon. Note that both ‘sense’ and ‘antisense’ dsRNA templates are generated, from which sense and antisense RNA strands are generated respectively, before being annealed to generate dsRNA. B, nucleotide sequence alignment of available Fasciola hepatica cathepsin L sequences, showing positioning of primers and their cross-reactivity across clades. Cathepsin L clades are indicated in sequence titles [CL1A, 1B, 2–6] as described elsewhere [67], [68]. This alignment was performed in mid 2010, these sequences represent those available in GenBank at that time, accession numbers refer to GenBank. (DOCX) [file pntd.0003185.s001.docx]

**Supporting Figure S1. *Fasciola hepatica* Cathepsin L (FheCatL) primer design**

**A. Layout of PCR oligonucleotides, dsRNA construct and qPCR amplicon:**

**PCR primers:**

**FheCatL FWD 1: TKRTTATGTGACGGAGGTGA**

**T7-FheCatL FWD 1: TAATACGACTCACTATAGGGTTKRTTATGTGACGGAGGTGA**

**FheCatL REV 2: GCCBKRTAHGGRTAAK**

**T7-FheCatL REV 2: TAATACGACTCACTATAGGGTGCCBKRTAHGGRTAAK**

**FheCatL REV 1: GTATAGAAGCCAGTCACTTTGGC**

dsRNA “sense” template = T7-FheCatL FWD 1 + FheCatL REV 2

dsRNA “antisense” template = FheCatL FWD 1 + T7-FheCatL REV 2

qPCR amplicon = FheCatL FWD 1 + FheCatL REV 1

**dsRNA (246 nt)**

**qPCR (309 nt)**

**B. FheCatL nucleotide sequence alignment for primer design:**

**____**

Z22767[FhCL5] ------------------------------------------------------------

EU287918[FhCL6] atttcatatgaagccgaaggcaatgacgtaccggccagtatagactggcgtcaata**tggt**

EU287915[FhCL3] atttcatatgaggccgaaggcaatgacgtcccggccagtatagactggcgtcaata**tggt**

AJ279091[FhCL3] gtttcatatgaagccgaaggcaatgacgtaccggccagtatagattggcgtgaata**tggt**

EU191984[FhCL3] atttcatatgaagccgaaggcaatgacgtaccggccagtatagactggcgtcaata**tggt**

AJ279093[FhCL3] atttcatatgaagcggaaggcaaagacgtaccggccagtatagactggcgtcaata**tggt**

EU287914[FhCL3] atttcatatgaagccgaaggcaatgacgtaccggccagtgtagattggcgtgaata**tggt**

EU195859[FhCL3] atttcatatgaagccgaaggcaatgacgtaccggccagtatagactggcgtcaata**tggt**

DQ534446[FhCL3] atttcatatgaagccgaaggcaatgacgtaccggccagtatagactggcgtcaata**tggt**

EU287917[FhCL4] atcccgtatgaggcaaacgatcgtgccgtacccgagagtattgattggcgtgaatt**tggt**

EU287916[FhCL4] atcccgtatgaggcaaacgatcgtgccgtacccgagagtattgattggcgtgaatt**tggt**

Z22763[FhCL1B] ------------------------------------------------------------

DQ533985[FhCL2] -------------------------acagaattcggttatgtgactggcgtgacta**ttat**

Z22764[FhCL2] ------------------------------------------------------------

EF407948[FhCL2] atcccgtataaggcgaacaagcttgccgtacccgagagcattgactggcgtgacta**ttat**

Z22765[FhCL2] atcccgtataaggcgaacaagcttgccgtacccgagagcattgactggcgtgacta**ttat**

EF611824[FhCL2] atcccgtataaggcgaacaagcttgccgtacccgagagcattgactggcgtgacta**ttat**

U62289[FhCL2] atcccgtttaaggcgaacaagcttgccgtacccgagagcattgactggcgtgacta**ttat**

Z22769[FhCL1B] ------------------------------------------------------------

AJ279092[FhCL1B] atcccgtatgaggcgaacaaccgtgccgtacccgacaaaattgactggcgtgaatc**tggt**

AY029229[FhCL1B] atcccgtatgaggcgaacaaccgtgccgtacccgacaaaattgactggcgtgaatc**tggt**

AB009306[FhCL1A] atcccgtatgaggcgaacaatcgtgccgtacccgacaaaattgactggcgtgaatc**tggt**

AY519972[FhCL1B] atcccgtatgaggcgaacaaccgtgccgtacccgacaaaattgactggcgtgaatc**tggt**

AY573569[FhCL1A] atcccgtatgaggcgaacaaccgtgccgtacccgacaaaattgactggcgtgaatc**tggt**

L33771[FhCL1A] gtcccgtatgaggcgaacaatcgtgccgtacccgacaaaattgactggcgtgaatc**tggt**

AF490984[FhCL1A] gtcccgtatgaggcgaacaatcgtgccgtacccgacaaaattgactggcgtgaatc**tggt**

AY277628[FhCL1A] gtcccgtatgagacgaacaatcgtgccgtacccgacaaaattgactggcgtgaatc**tggt**

U62288[FhCL1A] gtcccgtatgaggcgaacaatcgtgccgtacccgacaaaattgactggcgtgaatc**tggt**

AY519971[FhCL1A] gtcccgtatgagacgaacaatcgtgccgtacccgacaaaattgactggcgtgaatc**tggt**

DQ533986[FhCL1B] -------------------------acagaattcggctatgtgactggcgtgaatc**tggt**

Z22766[FhCL5] ------------------------------------------------------------

L33772[FhCL5] atcccatataaggctaacaagcgtgctgtacccgacagaattgactggcgtgaatcc**ggt**

AF271385[FhCL5] atcccatataaggctaacaagcgtgctgtacccgacagaattgactggcgtgaatcc**ggt**

**TKRT**

**FheCatL dsRNA F**

Z22767[FhCL5] ---------------------caggggcagtgcgggacgtgttgggcgttcgcaaccaca

EU287918[FhCL6] **tatgtgacggaggtga**aagatcagggagggtgtggttcctgttgggctttttcaaccacc

EU287915[FhCL3] **tatgtgacggaggtga**aagatcagggacaatgtggttcctgttgggctttttcagccgtc

AJ279091[FhCL3] **tatgtgacggaggtga**aagatcagggacaatgtggttcctgttgggctttttcagccgtc

EU191984[FhCL3] **tatgtgacggaggtga**aagatcagggacaatgtggttcctgttgggctttttcagccgtc

AJ279093[FhCL3] **tatgtgacggaggtga**aagatcagggacaatgtggttcctgttgggctttttcacccgtc

EU287914[FhCL3] **tatgtgacggaggtga**aagatcagggacaatgtggttcctgttgggctttttcagccgtc

EU195859[FhCL3] **tatgtgacggaggtga**aagatcagggacaatgtggttcctgttgggctttttcagccgtc

DQ534446[FhCL3] **tatgtgacggaggtga**aagatcagggacaatgtggttcctgttgggctttttcagccgtc

EU287917[FhCL4] **tatgtgac**c**gaggtga**aagatcagggagactgtggatcctgttgggcattctcaacaacc

EU287916[FhCL4] **tatgtgac**c**gaggtga**aagatcagggagactgtggatcctgttgggcattctcaacaacc

Z22763[FhCL1B] ---------------------caggggcaatgtgggtggtgttgggctttctcaacaaca

DQ533985[FhCL2] **tatgtgac**t**gaggtga**aagatcagggacaatgtggttcctgttgggctttctcaacaacc

Z22764[FhCL2] ---------------------caagggcaatgtgggtggtgttgggctttctcaacaacc

EF407948[FhCL2] **tatgtgac**t**gaggtga**aaaatcagggacaatgtggttcctgttgggctttctcaacaacc

Z22765[FhCL2] **tatgtgac**t**gaggtga**aagatcagggacaatgtggttcctgttgggctttctcaacaacc

EF611824[FhCL2] **tatgtgac**t**gaggtga**aagatcagggacaatgtggttcctgttgggctttctcaacaacc

U62289[FhCL2] **tatgtgac**t**gaggtga**aaaatcagggacaatgtggttcctgttgggctttctcaacaacc

Z22769[FhCL1B] ---------------------caggggcagtgtgggacgtgctgggcattctcaacaacc

AJ279092[FhCL1B] **tatgtgacgg**g**ggtga**aagatcagggaaactgtggttcctgttgggcattctcaacaacc

AY029229[FhCL1B] **tatgtgacggaggtga**aagatcagggaaactgtggttcctgttgggcattctcaacaacc

AB009306[FhCL1A] **tatgtgacggaggtga**aagatcagggaaattgtggttcatgttgggcattctcaacaacc

AY519972[FhCL1B] **tatgtgacgg**g**ggtga**aagatcagggaaactgtggttcctgttgggcattctcaacaacc

AY573569[FhCL1A] **tatgtgacgg**g**ggtga**aagatcagggaaactgtggttcctgttgggctttctcaacaacc

L33771[FhCL1A] **tatgtgacggaggtga**aagatcagggaaactgtggttcctgttgggcattctcaacaacc

AF490984[FhCL1A] **tatgtgacggaggtga**aagatcagggaaactgtggttcctgttgggcattctcaacaacc

AY277628[FhCL1A] **tatgtgacggaggtga**aagatcagggaaactgtggttcctgttgggcattctcaacaacc

U62288[FhCL1A] **tatgtgacggaggtga**aagatcagggaaactgtggctcctgttgggcattctcaacaacc

AY519971[FhCL1A] **tatgtgacggaggtga**aagatcagggaaactgtggttcctgttgggcattctcaacaacc

DQ533986[FhCL1B] **tatgt**a**acggaggtga**aagatcagggaaactgtggttcctgttgggctttctcaacaacc

Z22766[FhCL5] ---------------------caagggcagtgcgggaggtgttgggctttctcaacaaca

L33772[FhCL5] **tatgtgacggaggtga**aagatcagggaggctgtggttcttgttgggctttctcaacaaca

AF271385[FhCL5] **tatgtgacggaggtga**aagatcagggaggctgtggttcttgttgggctttctcaacaaca

**TATGTGACGGAGGTGA** ** ** ** ** ** ***** ** ** *

Z22767[FhCL5] ggggtcgttgaaggtcaatacagcagaaagtacgggagtaaaacaggattttcagaacaa

EU287918[FhCL6] ggtgcaattgagggacagtatgttaaaaagttccaaacccgagtgtcattctccgagcaa

EU287915[FhCL3] ggtgcaatagagggacagtatgtcaaaaagtttcaaaaccaaacgttattctccgagcaa

AJ279091[FhCL3] ggtgcaatagagggacagtatgtcaaaaagtttcaaaaccaaacgttattctccgagcaa

EU191984[FhCL3] ggtgcaatagagggacagtatgtcaaaaagtttcgaaaccgaatgttattctccgagcaa

AJ279093[FhCL3] ggtgcaatagaaggacagtatgtcaaaaagtttcaaaaccaaacgttattctccgagcaa

EU287914[FhCL3] ggtgcaatagagggacagtatctcagaaagtttcaaaaccaaacgttattctccgagcaa

EU195859[FhCL3] ggtgcaatagagggacagtatgtcaaaaagtttcaaaaccaaacgttattctccgagcaa

DQ534446[FhCL3] ggtgcaatagagggacagtatctcaaaaagtttcaaaaccaaacgttattctccgagcaa

EU287917[FhCL4] ggtgctgtggagggacaatatatgaaaaacccaaaagctaacatttccttttctgaacaa

EU287916[FhCL4] ggtgctgtggagggacaatatacgaaaaaccaaaaagctaacatttctttttctgaacaa

Z22763[FhCL1B] ggagctctggaaggacagtacatgaaaagtcagaggattaatatttcattctctgaacaa

DQ533985[FhCL2] ggtgctgtggagggacagtttaggaagaacgaaagagctagtgcttcattctctgagcaa

Z22764[FhCL2] ggtgctgtggagggacagtttaggaagaacgaaagagctagtgcttcattctctgagcaa

EF407948[FhCL2] ggtgctgtggagggacagtttaggaagaacgaaagagctagtgcttcattctctgagcaa

Z22765[FhCL2] ggtgctgtggagggacagtttaggaagaacgaaagagctagtgcttcattctctgagcaa

EF611824[FhCL2] ggtgctgtggagggacagtttaggaagaacgaaagagctagtgcttcattctctgagcaa

U62289[FhCL2] ggtgctgtggagggacagtttaggaagaacgaaagagctagtgcttcattctctgagcaa

Z22769[FhCL1B] ggtactatggagggacagtatatgaaaaagcagagaactagtatttcattctctgacgaa

AJ279092[FhCL1B] ggtactatggagggacaatatatgaaaaacgaaaaaactagtatttcattctctgagcaa

AY029229[FhCL1B] ggtactatggagggacaatatatgaaaaacgaaagaactagtatttcattctctgagcaa

AB009306[FhCL1A] ggtactatggaaggacagtatatgaaaaacgaaagaactagtatttcattctctgagcaa

AY519972[FhCL1B] ggtactatggagggacaatatatgaaaaacgaaagaactagtatttcattctctgagcaa

AY573569[FhCL1A] ggtactacggagggacagtatatgaaaaacgagagaactagtatttcattctctgagcaa

L33771[FhCL1A] ggtactatggagggacagtatatgaaaaacgaaagaactagtatttcattctctgagcaa

AF490984[FhCL1A] ggtactatggagggacagtatatgaaaaacgaaagaactagtatttcattctctgagcaa

AY277628[FhCL1A] ggtactatggagggacagtatatgaaaaacgaaagaactagtatttcattctctgagcaa

U62288[FhCL1A] ggtactatggagggacagtatatgaaaaacgaaagaactagtatttcattctctgagcaa

AY519971[FhCL1A] ggtactatggagggacagtatatgaaaaacgaaagaactagtatttcattctctgagcaa

DQ533986[FhCL1B] ggtactatgaagggacagtatatgaaaaacgagagaactagtatttcattctctgagcaa

Z22766[FhCL5] ggtgctacggaaggacagtatatgaaaaaccaaagaactagtatttcattctctgaacaa

L33772[FhCL5] ggtgctatggaaggacagtatatgaaaaacgaaaaaactagtatttcattctctgagcaa

AF271385[FhCL5] ggtgctatggaaggacagtatatgaaaaaccaaagaactagtatttcattctctgaacaa

** * ** ** * * * ** ** ** **

Z22767[FhCL5] caactggttgactgccgtcgtcggcacggcaacgaaggttgtaacggtggtctgatgact

EU287918[FhCL6] cagttggttgactgtagcacaatacctggaaaccacggatgtcgaggtggaggaatgagg

EU287915[FhCL3] cagttggtcgatcgtaccagaagatttggcaaccacggatgtggaggtggttggatggag

AJ279091[FhCL3] cagttggtcgattgtaccagaagatttggcaaccacggatgtggaggtggttggatggag

EU191984[FhCL3] cagttggtcgattgtaccaaacgatttggcaaccacggatgtagcggtggttggatggag

AJ279093[FhCL3] caattggtcgattgtaccagaagatttggcaaccacggatgtggaggtggttggatggag

EU287914[FhCL3] cagttggtcgattgtaccagaagatttggcaaccacggatgtggaggtggttggatggag

EU195859[FhCL3] cagttggtcgattgtaccagaagatttggcaaccacggatgtggaggtggttggatggag

DQ534446[FhCL3] cagttggtcgattgtaccagaagatttggcaaccacggatgtggaggtggttggatggag

EU287917[FhCL4] caattggttgattgtagtggtgattatggcaatcatggttgtaatggtggattcatggaa

EU287916[FhCL4] caattggttgattgtagtggtgattatggcaatcatggttgtaatggtggattcatggaa

Z22763[FhCL1B] caactggtcgattgtagcggtgattttggtaatcatggttgtagtggtggattaatggaa

DQ533985[FhCL2] caactggtcgattgtacccgtgattttggcaattatggttgcggtggaggatatatggaa

Z22764[FhCL2] caactggtcgattgtacccgtgattttggcaattatggttgcggtggaggatatatggaa

EF407948[FhCL2] caactggtcaattgtacccgtgattttggcaattatggttgcggtggaggatatgtggaa

Z22765[FhCL2] caactggtcgattgtacccgtgattttggcaattatggttgcggtggaggatatatggaa

EF611824[FhCL2] caactggtcgattgtacccgtgattttggcaattatggttgcggtggaggatatatggaa

U62289[FhCL2] caactggtcgattgtccccgtgatttgggcaattatggttgcggtggaggatatatggaa

Z22769[FhCL1B] caactggtcgattgtagccgtccttggggaaataatggttgcggtggtgggttgatggaa

AJ279092[FhCL1B] caactggtcgattgtagcggtccttggggaaataatggttgcagtggtggattgatggaa

AY029229[FhCL1B] caactggtcgattgtagcggtccttggggaaataatggttgcagtggtggattgatggaa

AB009306[FhCL1A] caactggtcgattgtagcggtccttggggaaattatggttgcatgggcggattgatggaa

AY519972[FhCL1B] caactggtcgattgtagcggtccttggggaaataatggttgcagtggtggattgatggaa

AY573569[FhCL1A] caactggtcgattgtagcggtccttggggaaataatggttgcggtggtggattgatggaa

L33771[FhCL1A] caactggtcgattgtagtggtccttggggaaataatggttgcagtggtggattgatggaa

AF490984[FhCL1A] caactggtcgattgtagtggtccttggggaaataatggttgcagtggtggattgatggaa

AY277628[FhCL1A] caactggtcgattgtagcggtccttggggaaataatggttgcagtggtggattgatggaa

U62288[FhCL1A] caactggtcgattgtagccgtccttggggaaataatggttgcggtggtggattgatggaa

AY519971[FhCL1A] caactggtcgattgtagtggtccttggggaaataatggttgcagtggtggattgatggaa

DQ533986[FhCL1B] caactggtcgattgtagccgtccttggggaaataatggttgcggtggtggattaatggaa

Z22766[FhCL5] caactggtcgattgtagccgtgattttggcaattatggttgtaatggtggactaatggaa

L33772[FhCL5] caactggtcgattgtagcggtccttttggcaattatggttgtaatggtggactaatggaa

AF271385[FhCL5] caactggtcgattgtagccgtgattttggcaattatggttgtaatggtggactaatggaa

** **** * * ** ** * ** ** ** ** **

**FheCatL dsRNA R**

Z22767[FhCL5] agttcctatcgatatttgatgaataactcgttggaatcagaaggtg**a**c**tatccatat**g**ag**

EU287918[FhCL6] agagcctatgagtatttgaaaaagaacggattggaaccggaatcct**cttatccgtacaag**

EU287915[FhCL3] aacgcttataaatatttgaaaaacagcggattggaaacggcatcct**attacccgtat**c**ag**

AJ279091[FhCL3] aacgcttataaatatttgaaaaacagcggattggaaacggcatcct**attacccgtat**c**ag**

EU191984[FhCL3] aacgcatatagatatttgaaagacagcggattggaaacggcatcct**attacccgtat**c**ag**

AJ279093[FhCL3] aacgcttataaatatttgaaaaacagcggattggaaacggcatccg**attacccgtat**c**ag**

EU287914[FhCL3] aacgcttataaatatttgaaaaacagcggattggaaacggcatccg**attacccgtat**c**ag**

EU195859[FhCL3] aacgcttataaatatttgaaaaacagcggattggaaacggcatcct**attacccgtat**c**ag**

DQ534446[FhCL3] aacgcttataaatatttgaaaaacagcggattggaaacggcatcct**attacccgtat**c**ag**

EU287917[FhCL4] aatgcttacgaatatctggaaagaaggggattagaaaccgaatctt**cttatccgtac**a**ag**

EU287916[FhCL4] aatgcttacgaatatctggaaagaaggggattagaaaccgaatctt**cttatccgtac**a**ag**

Z22763[FhCL1B] aaggcttacgaatatttgcgacatttcggattggaaacggaatcct**c**c**tat**t**cgtac**a**ga**

DQ533985[FhCL2] aacgcttatgaatatttgaaacacaacggattggaaactgagtcct**attatccatac**c**ag**

Z22764[FhCL2] aacgcttatgaatatttgaaacacaacggattggaaactgagtcct**attatccatac**c**ag**

EF407948[FhCL2] aacgcttatgaatatttgaaacacaacggattggaaactgagtcct**attatccatac**c**ag**

Z22765[FhCL2] aacgcttatgaatatttgaaacacaacggattggaaactgagtcct**attatccatac**c**ag**

EF611824[FhCL2] aacgcttatgaatatttgaaacacaacggattggaaactgagtcct**attatccatac**c**ag**

U62289[FhCL2] aacgcttatgaatatttgaaacacaacggattggaaactgagtcct**attatccatac**c**ag**

Z22769[FhCL1B] aatgcttaccaatatttgaaacaatttggattggaaaccgaatcct**cttatccgtac**a**cg**

AJ279092[FhCL1B] aatgcatacgaatatttgaaacgatttggattggaaaccgagtctt**cttatccctac**a**gg**

AY029229[FhCL1B] aatgcttaccaatatttgaaacaatttggattggaaaccgaatcct**cttatccgtac**a**cg**

AB009306[FhCL1A] aatgcttacgaatatttgaaacaatttggattggaaaccgaatcct**cttatccgtac**a**cg**

AY519972[FhCL1B] aatgcttaccaatatttgaaacaatttggattggaaaccgaatcct**cttatccgtac**a**cg**

AY573569[FhCL1A] aatgcatatgaatatttgaaacaatttggattggaaaccgaatcct**cttatccgtac**a**cg**

L33771[FhCL1A] aatgcttaccaatatttgaaacaatttggattggaaaccgaatcct**cttatccgtac**a**cg**

AF490984[FhCL1A] aatgcttaccaatatttgaaacaatttggattggaaaccgaatcct**cttatccgtac**a**cg**

AY277628[FhCL1A] aatgcttaccaatatttgaaacaatttggattggaaaccgaatcct**cttatccgtac**a**cg**

U62288[FhCL1A] aatgcttaccaatatttgaaacaatttggattggaaaccgaatcct**cttatccgtac**a**cg**

AY519971[FhCL1A] aatgcttaccaatatttgaaacaatttggattggaaaccgaatcct**cttatccgtac**a**cg**

DQ533986[FhCL1B] aatgcttacgaatatttgaaacaatttggattggaaaccgaatctt**cttatccgtac**a**gc**

Z22766[FhCL5] aatgcatacgaatatttgaaacgatttggattggaaaccgagtctt**cttatccttac**a**gg**

L33772[FhCL5] aatgcatacgaatatttgaaacgatttggattggaaaccgagtctt**cttatccttac**a**gg**

AF271385[FhCL5] aatgcatacgaatatttgaaacgatttggattggaaaccgagtctt**cttatcctta**ca**gg**

* * ** *** ** ** *** * * ** * **

**FheCatL qPCR R**

Z22767[FhCL5] **gc**catggacaacaggtgccgcgcgaaccgaactaagggtattgtgaaagttaaatcctat

EU287918[FhCL6] **gc**cgtggaaggtcagtgtcaatacaaaagcgatttggcacttgccaaagtgaccaatagc

EU287915[FhCL3] **gc**ttgggaatatccgtgtcaatacaggagagagcttggagtagccaaagtgactggtgcc

AJ279091[FhCL3] **gc**tgtggaatatcagtgtcaataccggaaagagcttggagtagccaaagtgactggtgcc

EU191984[FhCL3] **gc**ttgggaatatcaatgtcaatacaggagagagcttggagtagccaaagtgactggtgcc

AJ279093[FhCL3] **g**gttgggaatatcagtgtcaatacaggaaagagcttggagtagccaaagtgactggtgcc

EU287914[FhCL3] **g**gttgggaatatcagtgtcaatacaggaaagagcttggagtagccaaagtgactggtgcc

EU195859[FhCL3] **g**gttgggaatatcagtgtcaatacaggaaagagcttggagtagccaaagtgactggtgcc

DQ534446[FhCL3] **g**gttgggaatatcagtgtcaatacaggaaagagcttggagtagccaaagtgactggtgcc

EU287917[FhCL4] **gc**tgaggaaggtccatgtaaatacgatagccggttgggagttgtcgaagtgtttggctac

EU287916[FhCL4] **gc**tgaggaaggtccatgtaaatacgatagccggttgggagttgtcgaagtgtttggctac

Z22763[FhCL1B] **gc**tgatgaaggtccgtgtcaatacgacaggcagttgggagttgcccaggtgtctggctat

DQ533985[FhCL2] **gc**tgtggaaggtccgtgtcaatacgatgggcggttggcatatgccaaagtgactggctac

Z22764[FhCL2] **gc**tgtggaaggtccgtgtcaatacgatgggcggttggcatatgccaaagtgactggctac

EF407948[FhCL2] **gc**tgtggaaggtccgtgtcaatacgatgggcggttggcatatgccaaagtgactggctac

Z22765[FhCL2] **gc**tgtggaaggtccgtgtcaatacgatgggcggttggcatatgccaaagtgactggctac

EF611824[FhCL2] **gc**tgtggaaggtccgtgtcaatacgatgggcggttggcatatgccaaagtgactggctac

U62289[FhCL2] **gc**tgtggaaggtccgtgtcaatacgatgggcggttggcatatgccaaagtgactggctac

Z22769[FhCL1B] **gc**tgtggaaggtcagtgtcgatacaatgagcagttaggagttgccaaagtgactggctac

AJ279092[FhCL1B] **gc**tgtggaaggacagtgtcgatacaacgagcagttgggagttgccaaagtgactggctac

AY029229[FhCL1B] **gc**tgtggaaggtcagtgtcgatacaataggcagttgggagttgccaaagtgactggctac

AB009306[FhCL1A] **gc**tgtggaaggtcagtgtcgatacaataggcagttgggagttgccaaagtgacggactac

AY519972[FhCL1B] **gc**tgtggaaggtcagtgtcgatacaataggcagttgggagttgccaaagtgactggctac

AY573569[FhCL1A] **gc**tgtagaaggtcagtgtcgacacagtaagcagttaggagttgccaaagtgactggctac

L33771[FhCL1A] **gc**tgtggaaggtcagtgtcgatacaataagcagttaggagttgccaaagtgactggctac

AF490984[FhCL1A] **gc**tgtggaaggtcagtgtcgatacaataagcagttaggagttgccaaagtgactggctac

AY277628[FhCL1A] **gc**tgtggaaggtcagtgtcgatacaataagcagttaggagttgccaaagtgactggctac

U62288[FhCL1A] **gc**tgtggaaggtcagtgtcgatacaataagcagttaggagttgccaaagtgactggcttc

AY519971[FhCL1A] **gc**tgtggaaggtcagtgtcgatacaatgagcagttaggagttgccaaagtgactggctac

DQ533986[FhCL1B] **gc**tgtggaaggtccgtgtcgatatgataggaagttgggagttgccaaagtgactggctac

Z22766[FhCL5] **gc**tgtggaaggacagtgtcgatacaacgagcagttgggagttgccaaagtgactggctac

L33772[FhCL5] **gc**tgtggaaggacagtgtcgatacaacgagcagttgggagttgccaaagtgactggctac

AF271385[FhCL5] **gc**tgtggaaggacagtgtcgatacaacgagcagttgggagttgccaaagtgactggctac

* ** ** * * * **

_____

Z22767[FhCL5] acagttctgaaaaatgaaagtgaaacgcactcaaggagctggtcgggtaccaggggtccg

EU287918[FhCL6] caattggtgcgttctggtaatgaaacgcaattaaagaacttgatcggtgcagaaggacct

EU287915[FhCL3] tatactgtgcattctggtgatgagatgaggttgatgcaaatggtcggtagagaaggacct

AJ279091[FhCL3] tatactgtgcattccggtgatgagatgaagttgatgccaatggtcggtagagaaggacct

EU191984[FhCL3] tatactgtgcattctggtgatgagatgaggttgatgcaaatggtcggtagagaaggacct

AJ279093[FhCL3] tatactgtgcattctggtgatgagatgaagttgatgccaatggtccgtaaaaaaggacct

EU287914[FhCL3] tatactgtgcattctggtgatgagatgaagttgatgcaaatggtcggtagagaaggacct

EU195859[FhCL3] tatactgtgcattctggtgatgagatgaagttgatgcaaatggtcggtagagaaggacct

DQ534446[FhCL3] tatactgtgcattctggtgatgagatgaagttgatgcaaatggtcggtagagaaggacct

EU287917[FhCL4] tttattgagcattctggtattgagtcaaaactagcgcatttagttggtgacaaaggacct

EU287916[FhCL4] tttattgagcattctggtattgagtcaaaactagcgcatttagttggtgacaaaggacct

Z22763[FhCL1B] tttattgtgcattctcaagatgaggtagcattgaagaatctaatcggtgtggaaggacct

DQ533985[FhCL2] tatactgtgcattctggcgatgagatagaattaaagaatttggtcggtaccgaaggacct

Z22764[FhCL2] tatactgtgcattctggcgatgagatagaattaaagaatttggtcggtaccgaagacctg

EF407948[FhCL2] tatactgtgcattctggcgatgagatagaattaaagaatttggtcggtaccgaaggacct

Z22765[FhCL2] tatactgtgcattctggcgatgagatagaattaaagaatttggtcggtaccgaagacctg

EF611824[FhCL2] tatactgtgcattctggcgatgagatagaattaaagaatttggtcggtaccgaaggacct

U62289[FhCL2] tatactgtgcattctggcgatgagatagaattaaagaatttggtcggtaccgaaggacct

Z22769[FhCL1B] tatactgtgcattctggcagtgaggtagaattgaaaaatctagtcggttccgaaggacct

AJ279092[FhCL1B] tatactgtgcattctggcagtgaggtagaattgaaaaatctagtcggttccgaaggacct

AY029229[FhCL1B] tatactgtgcattctggcagtgaggtagaattgaaaaatctagtcggttcccgaagacct

AB009306[FhCL1A] tatactgtgcattctggcagtgaggtagaattgaaaaatctagtcggtgccgaaggacct

AY519972[FhCL1B] tatactgtgcattctggcagtgaggtagaactgaaaaatctagtcggttccgaaggacct

AY573569[FhCL1A] tacactgtgcattctggcagtgaggtagaattgaaaaatctagtcggtgccgaaagacct

L33771[FhCL1A] tacactgtgcattctggcagtgaggtagaattgaaaaatctagtcggagcccgaagacct

AF490984[FhCL1A] tacactgtgcattctggcagtgaggtagaattgaaaaatctagtcggtgccgaaggacct

AY277628[FhCL1A] tacactgtgccttctggcagtgaggtagaattgaaaaatctagtcggtgccgaaggacct

U62288[FhCL1A] tatactgtgcattctggcagtgaggtagaattgaaaaatctagtcggtgccgaaggacct

AY519971[FhCL1A] tatactgtgcattctggcagtgaggtagaattgaaaaatctagtcggttccgaaggacct

DQ533986[FhCL1B] tatacggtacattctggagatgaggtagaattgcaaaatctagtcggtggcgaaggacct

Z22766[FhCL5] tatacggtacattctggagatgaggtagaattgcaaaatctagtcggtgccggaagacct

L33772[FhCL5] tatacggtacattctggcgatgaggtagaattgcaaaatctagtcggttgccgaagacct

AF271385[FhCL5] tatacggtacattctggagatgaggtagaattgcaaaatctagtcggtgccgaaggacct

*** * *
