# Supplementary material for: RNAi Dynamics in Juvenile Fasciola spp. Liver Flukes Reveals the Persistence of Gene Silencing In Vitro
Source: PLoS Negl Trop Dis. 2014 Sep 25;8(9):e3185. doi: 10.1371/journal.pntd.0003185 (PMC4177864; doi:10.1371/journal.pntd.0003185)
Supplement: Figure S2 — PCR primer design strategy for induction and detection of RNA interference (RNAi) in Fasciola hepatica μ-class glutathione transferase (GST) sequences. A, schematic layout of PCR primers used for generation of double stranded (ds)RNA templates labelled with T7 RNA polymerase promoter sequences, and a quantitative (q)PCR amplicon. Note that both ‘sense’ and ‘antisense’ dsRNA templates are generated, from which sense and antisense RNA strands are generated respectively, before being annealed to generate dsRNA. B, nucleotide sequence alignment of available Fasciola hepatica μ-GST sequences, showing positioning of primers and their cross-reactivity between sequences. GST titles and accession numbers are indicated. This alignment was performed in mid 2010, these sequences represent those available in GenBank at that time, accession numbers refer to GenBank. (DOCX) [file pntd.0003185.s002.docx]

**Supporting Figure S2. *Fasciola hepatica* μ-class glutathione transferase (FheμGST) primer design**

**A. Layout of PCR oligonucleotides, dsRNA construct and qPCR amplicon:**

**PCR primers:**

**FheμGST FWD 1: GGATAAGCATGGAATGCTTGGT**

**FheμGST FWD 2: ACACCCGAGGAACGAGCTCG**

**T7- FheμGST FWD 2: taatacgactcactatagggtACACCCGAGGAACGAGCTCG**

**FheμGST REV 1: TCGTAAACCATAAAGTCCACATGG**

**T7- FheμGST REV1: taatacgactcactatagggtTCGTAAACCATAAAGTCCACATGG**

dsRNA “sense” template = T7-FheμGST FWD 2 + FheμGST REV 1

dsRNA “antisense” template = FheμGST FWD 2 + T7- FheμGST REV 1

qPCR amplicon = FheμGST FWD 1 + FheμGST REV 1

**qPCR (243 nt)**

**dsRNA (218 nt)**

M77682.1_FHEGST51_muGST_ TGCAAACTGACTCAGTCGGTGGCCATAATGCGGTACATTGCGGACAAGCA 250

M93434.1_muGST_ TGCAAACTGACTCAGTCGGTGGCCATAATGCGGTACATTGCGGATAAGCA 210

M77680.1_FHEGST7_muGST_ TGCAAACTGACTCAATCGGTGGCCATAATGCGGTACATTGCGGATAAGCA 212

M77681.1_FHEGST47_muGST_ TGCAAACTGACTCAGTCGGTGGCCATAATGCGGTACATTGCGGACAAGCA 215

M77679.1_FHEGST1_muGST_ TGTAAACTAACTCAATCACTGGCCATATTGCGTTACATCGCCGATAAGCA 167

** ***** ***** ** ******** **** ***** ** ** *****

M77682.1_FHEGST51_muGST_ TGGAATGCTTGGTACCACACCCGAGGAACGAGCTCGAATTTCGATGATCG 300

M93434.1_muGST_ TGGAATGCTTGGTTCTACACCCGAGGAACGAGCTCGAATTTCGATGATCG 260

M77680.1_FHEGST7_muGST_ TGGAATGCTTGGTTCCACACCCGAGGAACGAGCTCGAATTTCTATGATCG 262

M77681.1_FHEGST47_muGST_ TGGAATGCTTGGTACCACACCCGAGGAACGAGCTCGAATTTCGATGATCG 265

M77679.1_FHEGST1_muGST_ CGGGATGATTGGTTCTACGCCCGAAGAACGAGCTCGAGTTTCGATGATCG 217

** *** ***** * ** ***** ************ **** *******

M77682.1_FHEGST51_muGST_ AAGGAGCTGCAATGGATCTTCGGATGGGTTTTGTTCGTGTTTGTTACAAC 350

M93434.1_muGST_ AAGGAGCTGCAATGGATCTTCGGATGGGTTTTGTTCGTGTTTGTTACAAC 310

M77680.1_FHEGST7_muGST_ AAGGAGCTGCAATGGATCTTCGGATAGGGTTTGGACTTACTTGCTACAAC 312

M77681.1_FHEGST47_muGST_ AAGGAGCTGCAATGGATCTTCGGATAGGGTTTGGACGTGTTTGCTACAAT 315

M77679.1_FHEGST1_muGST_ AAGGTGCTGCAGTAGATCTTCGTCAAGGCCTTTCCCGAATTTCTTACGAT 267

**** ****** * ******** ** ** * ** *** *

M77682.1_FHEGST51_muGST_ CCAAAATTTGAAGAAGTGAAAGGAGATTATCTGAAAGAACTGCCAACAAC 400

M93434.1_muGST_ CCAAAATTTGAAGAAGTGAAAGGAGATTATCTGAAAGAACTGCCAACAAC 360

M77680.1_FHEGST7_muGST_ CCAAAATTTGAAGAATTGAAAGGAGATTATTTGAAAGGACTGCCGACAAC 362

M77681.1_FHEGST47_muGST_ CCAAAATTTGAAGAAGTTAAGGAAGAGTATGTTAAAGAACTGCCCAAAAC 365

M77679.1_FHEGST1_muGST_ CCAAAATTTGAACAGCTGAAAGAAGGATACCTGAAGGACTTGCCGACAAC 317

************ * * ** * ** ** * ** * **** * ***

M77682.1_FHEGST51_muGST_ ATTGAAGATGTGGTCCAATTTTCTTGGAGATCGTCACTATTTGACAGGTT 450

M93434.1_muGST_ GTTGAAGATGTGGTCCGATTTTCTTGGAGATCGTCACTATTTGACAGGTT 410

M77680.1_FHEGST7_muGST_ GTTGAAGATGTGGTCCGATTTTCTCGGAGACCGTCAATATTTGATAGGTT 412

M77681.1_FHEGST47_muGST_ ATTGAAGATGTGGTCCGATTTTCTTGGAGATCGTCATTATTTGACAGGTT 415

M77679.1_FHEGST1_muGST_ GATGAAAATGTGGTCCGATTTTCTCGGTAAAAATCCTTACTTGAGAGGTA 367

**** ********* ******* ** * ** ** **** ****

M77682.1_FHEGST51_muGST_ CTCCAGTTAGCCATGTGGACTTTATGGTTTACGAAGCATTGGACTGTATT 500

M93434.1_muGST_ CTACAGTTAGCCATGTGGACTTTATGGTTTACGAAGCATTGGACTGTATT 460

M77680.1_FHEGST7_muGST_ CCTCAGTTAGCCATGTGGACTTTATGGTGTACGAAGCATTGGACTGTATT 462

M77681.1_FHEGST47_muGST_ CCTCAGTCAGCCATGTGGACTTCATGCTTTACGAAACACTGGATTCGATT 465

M77679.1_FHEGST1_muGST_ CCTCGGTAAGCCACGTCGATTTCATGGTGTACGAGGCTCTGGATGCGATT 417

* * ** ***** ** ** ** *** * ***** * **** ***
